# Senescent neutrophils-derived exosomal piRNA-17560 promotes chemoresistance and EMT of breast cancer via FTO-mediated m6A demethylation

**DOI:** 10.1038/s41419-022-05317-3

**Published:** 2022-10-27

**Authors:** Baochi Ou, Yuan Liu, Zongxuan Gao, Jun Xu, Yunwen Yan, Yongxiang Li, Jingjie Zhang

**Affiliations:** 1 Department of Breast Surgery, Department of General Surgery, The First Affiliated Hospital of Anhui Medical University, No. 218, Jixi Road, Hefei, 230022 Anhui China; 2 Department of General Surgery, Shanghai General Hospital, Shanghai Jiao Tong University, No. 85, Wujin Road, Shanghai, 200080 China; 3 Anhui Medical University, No. 218, Meishan Road, Hefei, 230022 Anhui China; 4 Department of General Surgery, The First Affiliated Hospital of Anhui Medical University, No. 218, Jixi Road, Hefei, 230022 Anhui China

**Keywords:** Cancer microenvironment, Senescence

## Abstract

Cellular senescence is characterized by a tumor-suppressive program as well as a pro-inflammatory secretome. Neutrophils constitute significant compositions of malignancies and play key roles in tumor development. However, the role of senescent neutrophils in cancer progression is presently unexplored. Here, we demonstrate that neutrophils display enhanced senescence in breast cancer patients receiving chemotherapy. The senescent neutrophils produce increased number of exosomes, which confer drug resistance to tumor cells in vitro and in vivo. Mechanistically, senescent neutrophils-derived exosomal piRNA-17560 enhances the expression of fat mass and obesity-associated protein (FTO) in breast cancer cells. The upregulation of FTO further strengthens ZEB1 transcripts stability and expression by decreasing N6-methyladenosine (m6A) RNA methylation, leading to chemoresistance and epithelial-mesenchymal transition (EMT) of tumor cells. Clinically, the level of exosomal piR-17560 correlates with poor chemotherapy response in patients with breast cancer. In addition, YTHDF2 is essential for the posttranscriptional regulation of ZEB1 by piRNA-17560/FTO signaling. Senescent neutrophils secret exosomal piR-17560 in a STAT3-dependent manner. Altogether, this study suggests that senescent neutrophils-derived exosomal piR-17560 confers chemoresistance to tumor cells and senescent neutrophils may serve as a potential therapeutic target in breast cancer.

## Introduction

Breast cancer (BC) is the most commonly diagnosed cancer worldwide in 2020, with 2.3 million new cases and over 690,000 deaths recorded [1]. Despite recent progress in this field, clinical challenges including chemoresistance and metastasis remain to be settled.

Cellular senescence refers to a program of irreversible cell cycle arrest that can be triggered by a number of oncogenic stresses and has therefore been considered to suppress carcinogenesis [2, 3]. Senescent cells generally exhibit morphological alterations including enlarged cell size with increased stress granules and are characterized by the expression of senescence-associated beta-galactosidase (SA-βGal), p53, cyclin-dependent kinase inhibitor p16^INK4A^ and p21^CIP1^ [4]. Interestingly, it becomes gradually obvious that senescent cells are not just non-proliferative but also produce a plethora of inflammatory cytokines, chemokines and proteases. This novel feature, termed the senescence-associated secretory phenotype (SASP), exerts various biological effects on cellular homeostasis [5, 6]. By means of the SASP secretion, senescent cells are capable of remodeling the local environment and communicating with the immune cells in aging sites [7]. Therefore, studying pathological and physiological roles of the SASP may provide a better understanding of senescence-associated diseases, such as cancer.

Neutrophils are the most prominent leukocyte in circulation and have essential roles in inflammatory responses. In recent years, they are also recognized as a component of the immune system to mediate tumor progression. However, the exact function of neutrophils in malignancies is still controversial as they were described to possess both pro- and anti-tumorigenic effects [8]. Our recent investigation identified a novel neutrophil subset, C5aR1-positive neutrophils, which can drive breast cancer glycolysis [9]. Although it has been reported that neutrophils facilitate cellular senescence in a model of acute liver injury [10], it remains unknown whether neutrophils undergo senescence and whether the senescent neutrophils play a role during tumor progression.

In this study, we demonstrate that senescent neutrophils, which abundantly reside in therapy-treated tissues, can confer resistant phenotype to recipient cancer cells by the generation of exosomes. We find that the exosomal PIWI-interacting RNA-17560 (piRNA-17560) secreted by senescent neutrophils is responsible for chemoresistance and epithelial-mesenchymal transition (EMT) of BC cells through a N6-methyladenosine (m6A)-dependent mechanism. Our findings suggest a novel avenue to improve therapeutic efficacy by harnessing senescent neutrophils-derived exosomes.

## Results

### Neutrophils display enhanced senescence in BC patients receiving chemotherapy with increased secretion of exosomes

**Fig. 1:**
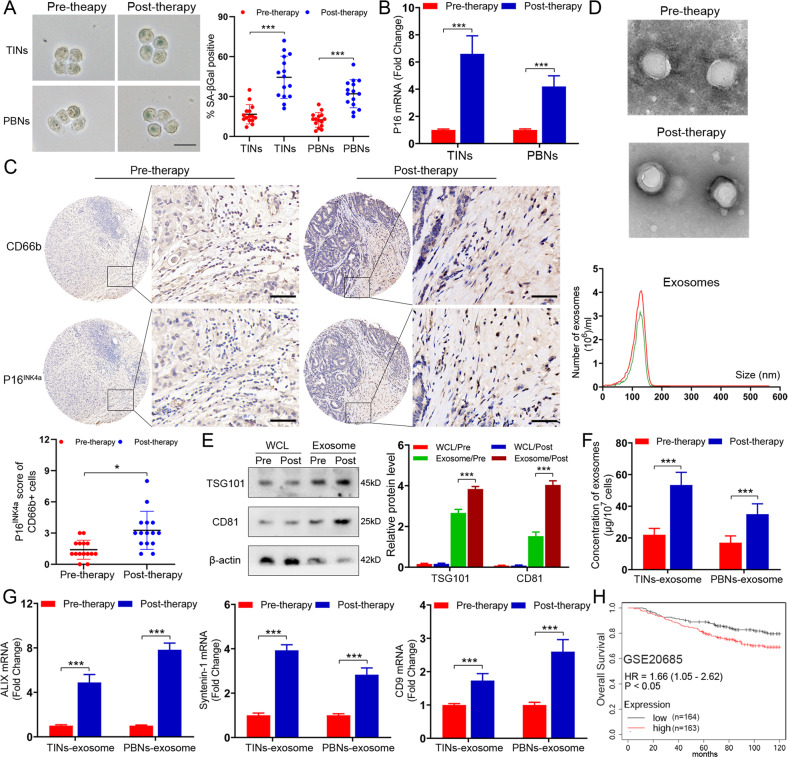
Neutrophils exhibit high senescence in BC patients receiving chemotherapy with increased generation of exosomes. **A** Representative image of SA-βGal staining in the TINs and PBNs extracted from BC patients (*n* = 15) before and after chemotherapy. Scare bars, 20 μm. **B** qPCR analysis of P16^INK4a^ in the TINs and PBNs extracted from BC patients before and after chemotherapy. **C** Representative images of P16^INK4a^ and CD66b staining in BC patients before and after chemotherapy (above). Scare bars, 50 μm. The P16^INK4a^ staining score of CD66b^+^ cells in tumors pre- and post-chemotherapy (below). **D** Phenotype analysis of exosomes derived from pre-therapeutic and post-therapeutic TINs using electron microscopy and Nano Sight nanoparticle tracking analysis. **E** Immunoblotting of TSG101 and CD81 in whole cell lysates (WCL) and exosomes of pre-therapeutic and post-therapeutic TINs. Densitometry represents the expression of the proteins relative to β-actin. **F** The number of exosomes in the exosome fraction was quantified in pre-therapeutic and post-therapeutic neutrophils. **G** qPCR analysis of ALIX, Syntenin-1, and CD9 in the exosomes of the TINs and PBNs extracted from BC patients before and after chemotherapy. **H** Kaplan–Meier survival analysis showing overall survival based on the expression of senescent neutrophil gene signature in GSE20685. Data represent the mean ± SD of at least three independent experiments. *P < 0.05, **P < 0.01, ***P < 0.001.

To investigate whether neutrophils undergo senescence during chemotherapy, we extracted neutrophils from primary tumor tissues (named TINs) and peripheral blood (named PBNs) of patients before and after neoadjuvant chemotherapy. Intriguingly, senescence-associated betagalactosidase (SA-βGal) staining showed clearly visible signals in the neutrophils of posttreatment patients, in contrast to the samples from pretreatment ones (Fig. 1A). Moreover, both TINs and PBNs displayed elevated expression levels of p16^INK4a^ in the posttreatment patients, as compared with those pretreatment individuals (Fig. 1B). In addition, by using IHC staining, we observed enhanced p16^INK4a^ expression in TINs from the patients who experienced chemotherapeutic intervention (Fig. 1C). Since senescent cells always produce increased number of exosomes, we then examined if the secretion of exosomes was increased in the neutrophils from BC patients undergoing chemotherapy. Exosomes were isolated from TINs and exhibited a typical exosomal structure and size of ~50–150 nm (Fig. 1D). Further immunoblotting analysis confirmed the presence of exosome-markers, tumor susceptibility gene 101 (TSG101) and CD63 (Fig. 1E). As expected, the TINs from post-therapy patients secreted a higher concentration of exosomes compared with pre-therapy ones (Fig. 1F). There was a concomitant upregulation of the majority of exosome-associated molecules (ALIX, Syntenin-1 and CD9), as indicated by qPCR assays (Fig. 1G). Similar results were also found in the PBNs from patients before and after therapy (Fig. 1F, G). To explore the clinical relevance of senescent neutrophils, we generated a gene signature (CD66b, p16^INK4a^, p21^CIP1^, and TP53) to examine the abundance of senescent neutrophils in BC. The high senescent neutrophil gene signature group had a worse overall survival than the low group, as shown in GSE20685 and GSE65194 (Fig. 1H and Supplementary Fig. 1A) [11, 12]. Altogether, neutrophils exhibit increased senescence with elevated secretion of exosomes in BC patients receiving chemotherapy.

### Senescent neutrophils promote the proliferation and chemoresistance of BC cells via exosomes

**Fig. 2:**
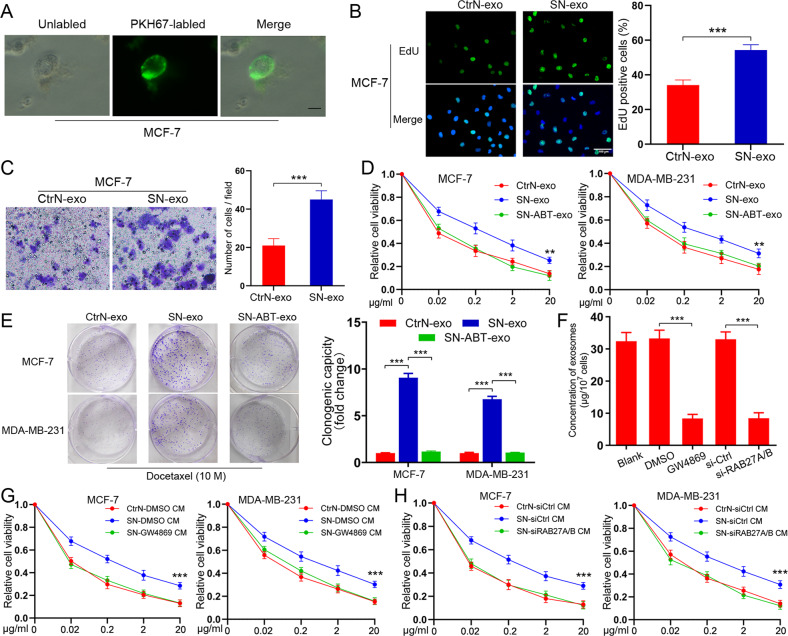
Senescent neutrophils secret exosomes to promote the proliferation and chemoresistance of BC cells. **A** Representative confocal microscopy image showing the internalization of PKH67-labeled exosomes (green) by MCF-7 cells. Scare bars, 10 μm. **B** Cell proliferation as measured by EdU incorporation was promoted by SN-exo in MCF-7 cells. Scare bars, 100 μm. **C** Migration ability of MCF-7 cells was enhanced by SN-exo, tested by transwell assay. **D** CCK8 assay of BC cells pre-incubated with indicated exosomes for 48 h followed by docetaxel treatment at indicated concentrations for 48 h. **E** Colony formation assay of BC cells pre-incubated with indicated exosomes for 48 h followed by docetaxel at indicated concentrations for 48 h. **F** Quantification of the number of exosomes from senescent neutrophils with indicated treatments. **G**, **H** CCK8 assay of BC cells pre-incubated with indicated CM for 48 h followed by docetaxel at different concentrations for 48 h. Data represent the mean ± SD of at least three independent experiments. *P < 0.05, **P < 0.01, ***P < 0.001.

To study the role of exosomes derived from senescent neutrophils, we treated human PBNs and HL-60 neutrophils with a pre-optimized sublethal dose of doxorubicin, an agent frequently used in breast cancer clinics. The agent resulted in enhanced SA-βGal staining positivity, elevated DNA damage foci, and upregulation of multiple SASP factors (CSF3, CCL3, CXCL8, and IL1α) 48 hours later (Supplementary Fig. 1B–D). We then incubated MCF-7 cells with the exosomes purified from doxorubicin-induced senescent PBNs or control pre-senescent neutrophils (named CtrN). Notably, MCF-7 cells effectively engulfed PKH67-labeled exosomes (Fig. 2A). The senescent neutrophils-derived exosomes (named SN-exo) significantly promoted MCF-7 cell proliferation and migration (Fig. 2B, C). Given cellular senescence usually occurs upon therapeutic stimuli, we then determined if senescent neutrophils-secreted exosomes played a role in drug response of cancer cells. Interestingly, SN-exo-cultured MCF-7 and MDA-MB-231 were relatively more resistant to docetaxel (a commonly used agent in clinics) than those cultured with CtrN-exo (Fig. 2D). The coculture colony formation assay also confirmed that SN-exo could confer the resistant phenotype to recipient BC cells (Fig. 2E). When using ABT-36 to remove senescent neutrophils from doxorubicin-treated PBNs, we found that the extracted exosomes (named SN-ABT-exo) largely abolished the effect of SN-exo (Fig. 2D, E). We further examined the response of recipient cells to doxorubicin. Similarly, compared to CtrN-exo-cultured cells, SN-exo-treated tumor cells showed reduced sensitivity to doxorubicin (Supplementary Fig. 1E). Next, we reduced exosome secretion via the pharmacological targeting of neutral sphingomyelinase-2 (nSMase) with GW4869 or RAB27A/B knockdown with siRNA (Supplementary Fig. 1F). As expected, exosome number in the culture medium (CM) was remarkably decreased by nSMase inhibition or RAB27A/B silencing (Fig. 2F), with no influence on neutrophils viability (Supplementary Fig. 1G). Importantly, the CM from senescent neutrophils with GW4869 treatment (Fig. 2G) or RAB27A/B knockdown (Fig. 2H) failed to confer docetaxel resistance to recipient cells, suggesting the key role of SN-exo for the transfer of drug resistance.

### Senescent neutrophils enhance the chemoresistance of BC cells through exosomal piR-17560

**Fig. 3:**
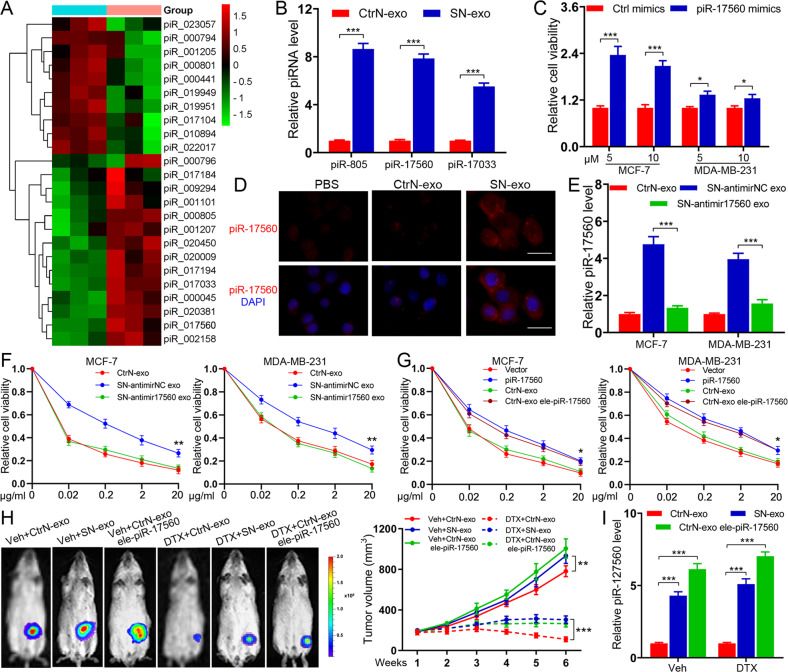
Senescent neutrophils confer docetaxel resistance to BC cells through exosomal piR-17560. **A** Heatmap diagram of differential piRNA expression in senescent neutrophils-derived exosomes and control exosomes. Red, increased expression; green, decreased expression. **B** qPCR analysis of piRNA expression in the exosomes produced from control and senescent neutrophils. **C** The viability of cancer cells transfected with piRNA mimics followed by docetaxel at different concentrations for 48 h. **D** Representative image of immunofluorescence staining showing the expression of piR-17560 in MCF-7 cells treated with indicated exosomes (PBS as control) for 48 h. Scare bars, 50 μm. **E** qPCR analysis of piR-17560 expression in tumor cells cultured with indicated exosomes for 48 h. **F** CCK8 assay of cancer cells pre-incubated with indicated exosomes for 48 h followed by docetaxel at different concentrations for 48 h. **G** CCK8 assay of cancer cells transfected with piR-17560 plasmids or incubated with indicated exosomes followed by docetaxel administration for 48 h. **H** In vivo xenograft assay of MCF-7 cells in nude mice with intratumoral injection of indicated exosomes upon docetaxel (10 mg/kg) or vehicle treatment. Representative bioluminescent images of mice in indicated groups are shown (left). Tumor volumes are shown (*n* = 5 per group, right). **I** qPCR analysis of piR-17560 expression in each group of xenografts. Data represent the mean ± SD of at least three independent experiments. *P < 0.05, ***P < 0.001.

To interrogate the underlying mechanisms for exosome-transferred chemoresistance, we performed small RNA-sequencing analysis of the exosomes from control and DXR-induced senescent neutrophils. The results showed a number of differentially expressed small RNAs, including piRNAs between two groups of exosomes (adjusted *p*-value < 0.05 and fold change > 2). We then examined the piRNA profiles (Fig. 3A) and chose the top 10 dysregulated piRNAs (5 upregulated and 5 downregulated) for further investigation. qPCR analysis showed that the expression levels of three piRNAs (piR-805, piR-17560, and piR-17033) were markedly increased in SN-exo (Fig. 3B). Moreover, we observed that only piR-17560 mimics conferred the resistant phenotype to recipient BC cells, whereas the other two piRNAs had no such effects (Fig. 3C and Supplementary Fig. 2A). The increase of piR-17560 levels in recipient cells was not affected by actinomycin D (Supplementary Fig. 2B), excluding the involvement of endogenous induction. RNA fluorescence in situ hybridization (FISH) indicated that piR-17560 was localized in both the nucleus and cytoplasm of recipient cells (Fig. 3D). To further explore the functional role of piRNA-17560, we utilized antagomirs to inhibit the generation of piRNA-17560 in senescent neutrophils (named SN-antimir17560). The SN-antimir17560-derived exosomes, which had lower expression of piRNA-17560 (Fig. 3E), were isolated and cocultured with tumor cells. We found that depletion of piR-17560 abolished the effect of SN-exo, increasing BC cells sensitivity to docetaxel (Fig. 3F). The preceding results raised a question concerning whether piR-17560 upregulation could sufficiently confer docetaxel tolerance to BC cells. As shown in Fig. 3G, forced expression of piR-17560 (Supplementary Fig. 2C) endowed tumor cells with refractoriness to docetaxel. The PBNs-derived exosomes electroporated with piR-17560 exhibited a similar effect (Fig. 3G), excluding the involvement of factors other than piR-17560 in exosomes. In addition, we observed a higher number of exosomal piR-17560 in senescent TINs (isolated from the patients who underwent therapy) relative to control TINs (Supplementary Fig. 2D). Senescent TINs-derived exosomal piR-17560 also confer drug resistance to breast cancer cells (Supplementary Fig. 2E).

To shed light on the effect of senescent neutrophils-derived piR-17560 on docetaxel response in vivo, we administered the exosomes intratumorally into MCF-7 xenografts. As shown in Fig. 3H–I, the exosomes extracted from senescent neutrophils significantly dampened the response of BC xenografts to docetaxel, accompanied by increased piR-17560 expression in the xenografts. Moreover, the exosomes electroporated with piR-17560 achieved a similar effect (Fig. 3H–I). To evaluate the possible clinical relevance of our findings, we isolated circulating exosomes from the plasma of BC patients. The results demonstrated a significantly higher expression of exosomal piR-17560 levels in BC patients than that in healthy donors (Supplementary Fig. 3A). Furthermore, the expression of circulating exosomal piR-17560 was significantly higher in BC patients with chemotherapy than in those without therapy (Supplementary Fig. 3A). Compared with matched pre-therapy plasma, the expression of exosomal piR-17560 was markedly increased in post-therapy plasma of patients receiving therapy (Supplementary Fig. 3B). Notably, the level of exosomal piR-17560 in post-therapy plasma was higher in patients who suffered from stable disease or progressive disease (SD + PD) during docetaxel-based neoadjuvant chemotherapy than in those with complete or partial regression (CR + PR). Thus, we conclude that exosomal piR-17560 may have a potential regulatory role in senescent neutrophils-mediated chemoresistance.

### Exosomal piR-17560 from senescent neutrophils promote the chemoresistance and EMT of BC cells via FTO/ZEB1 signaling

**Fig. 4:**
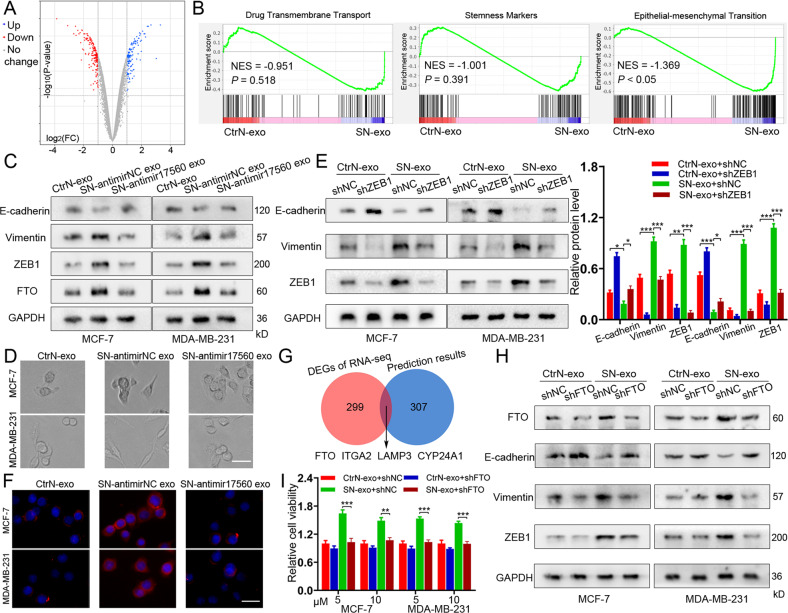
Exosomal piR-17560 from senescent neutrophils promote the chemoresistance and EMT of BC cells via FTO/ZEB1 signaling. **A** Volcano Plot showing the gene expression of MCF-7 cells cultured with senescent neutrophils-derived exosomes or control exosomes, examined by RNA sequencing. **B** Gene set enrichment analysis showing an enrichment of genes related to EMT in tumor cells cultured with SN-exo. Normalized enrichment score (NES) and false discovery rate (FDR) are indicated. **C** Immunoblotting of E-cadherin, Vimentin, ZEB1, and FTO in BC cells incubated with indicated exosomes. **D** The morphology of MCF-7 and MDA-MB-231 cells treated with indicated exosomes for 48 h. Scare bars, 50 μm. **E** Immunoblotting of E-cadherin, Vimentin and ZEB1 in BC cells with and without ZEB1 knockdown upon indicated exosomes treatment. **F** Immunofluorescence staining of Vimentin in MCF-7 and MDA-MB-231 cells treated with indicated exosomes. Scare bars, 50 μm. **G** Venn diagram showing the overlap of differentially expressed genes in SN-exo-cultured MCF-7 cells with a list of potential genes based on prediction algorithm. **H** Immunoblotting of FTO, E-cadherin, Vimentin, and ZEB1 in BC cells with and without FTO knockdown upon indicated exosomes treatment. **I** The viability of cancer cells with and without FTO knockdown upon indicated exosomes treatment followed by docetaxel for 48 h. Data represent the mean ± SD of at least three independent experiments. *P < 0.05, **P < 0.01, ***P < 0.001.

To better understand the effect of exosomal piR-17560 on tumor cells, we conducted RNA sequencing of MCF-7 cells that were treated with SN-exo or CtrN-exo. As expected, the mRNA profiling revealed a list of differentially expressed genes between two groups of cells (Fig. 4A and Table S1). It has been established that chemoresistance is related to drug efflux, stemness, epithelial-mesenchymal transition (EMT) and so on [13]. Using gene set enrichment analysis (GSEA), we found that only the “EMT” signatures were enriched in SN-exo-cultured cells (Fig. 4B). Among the EMT-associated genes, ZEB1 was the most upregulated one in SN-exo-treated MCF-7 cells. Thus, we speculated that piR-17560 from SN-exo might promote the chemoresistance and EMT of BC cells via the transcription factor ZEB1. Indeed, SN-exo transfer dramatically reduced E-cadherin expression and boosted the expression of Vimentin and ZEB1 (Fig. 4C and Supplementary Fig. 4A), rendered tumor cells a mesenchymal, spindle-like morphology (Fig. 4D). This effect was largely abolished by antimir-17560 transfection (Fig. 4C–D and Supplementary Fig. 4A), as well as the inhibition of ZEB1 in tumor cells (Fig. 4E and Supplementary Fig. 4B). Immunofluorescence staining of the mesenchymal marker Vimentin also confirmed this phenomenon in MCF-7 and MDA-MB-231 cells (Fig. 4F and Supplementary Fig. 4C). Considering piRNA exhibits imperfect complementarity with its mRNA targets, we then examined whether piR-17560 directly regulated ZEB1 expression by using a miRNA-specific target detection algorithm (miRanda). Intriguingly, piR-17560 had no putative binding sites within ZEB1 mRNA, suggesting an indirect modulation of ZEB1 by piR-17560.

To this end, we compared the RNA sequencing data with miRanda prediction results and identified that FTO, ITGA2, LAMP3, and CYP24A1 might be the potential bridge that connected piR-17560 to ZEB1 (Fig. 4G). Notably, there is one putative binding site of piR-17560 within the mRNA of FTO, but not other genes. This suggests FTO may be the target of piR-17560 that regulates ZEB1 expression. Moreover, we observed that SN-exo stimulation contributed to FTO expression, while the exosomes from piRNA-17560-depleted senescent neutrophils reduced both mRNA and protein levels of FTO (Fig. 4C and Supplementary Fig. 4D). Inhibition of FTO with shRNAs was sufficient to suppress the expression of ZEB1 and Vimentin, promote E-cadherin expression levels (Fig. 4H and Supplementary Fig. 4E), and attenuate docetaxel resistance of cancer cells following SN-exo incubation (Fig. 4I). Similarly, senescent TINs-derived exosomes could also promote the EMT (Supplementary Fig. 5A) and activate FTO/ZEB1 signaling in breast tumor cells (Supplementary Fig. 5B). In addition, high expression of FTO and ZEB1 was observed in the SN-exo-treated xenografts (Supplementary Fig. 5C). Both FTO and ZEB1 exhibited higher levels in patients who suffered from SD or PD than in those with CR or PR during DTX-based therapy (Supplementary Fig. 5D). In the breast cancer TCGA datasets, we found that the expression of ZEB1 was positively correlated with FTO (Supplementary Fig. 5E). Taken together, exosomal piR-17560 from senescent neutrophils can promote the chemoresistance and EMT of BC cells through activating FTO/ZEB1 axis.

### Senescent neutrophils-derived exosomal piR-17560 upregulates ZEB1 expression via FTO-mediated m6A demethylation

**Fig. 5:**
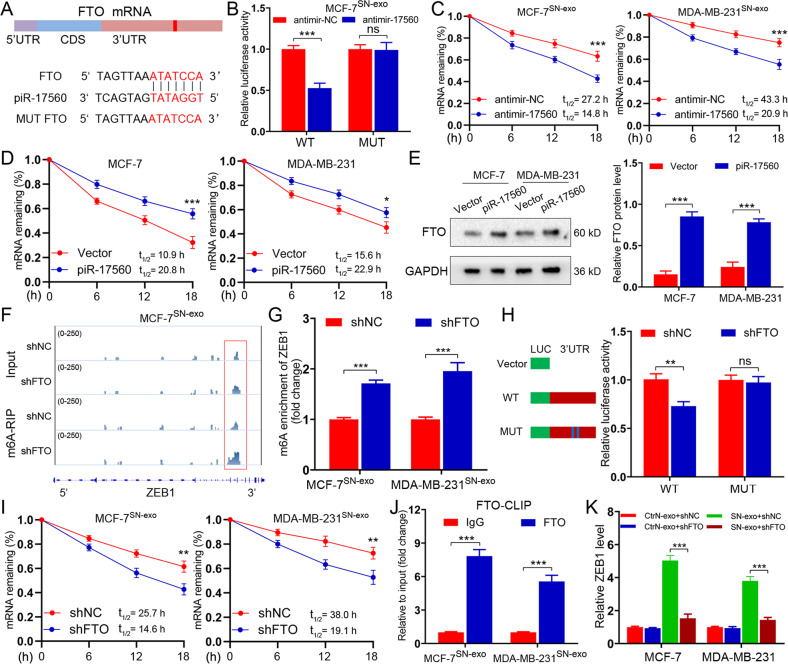
Senescent neutrophils secret exosomal piR-17560 to upregulate ZEB1 expression via FTO-mediated m6A modification. **A** A schematic representation of the interaction between piRNA-17560 and the 3’UTR of FTO. **B** Luciferase reporter assays showing the effect of piRNA-17560 on FTO reporters with either wild-type (WT) or mutated binding sites. **C** The indicated cells were transiently transfected with antimir-17560 or antimir-NC. The half-life (t1/2) of FTO mRNA was measured. **D** MCF-7 or MDA-MB-231 cells were transiently transfected with antimir-17560 or antimir-NC. The half-life (t1/2) of FTO mRNA was tested. **E** Immunoblotting of FTO in MCF-7 and MDA-MB-231 cells with and without piR-17560 overexpression. **F** m6A peaks were enriched at ZEB1 mRNA from m6A-RIP sequencing data of FTO-depleted MCF-7^SN-exo^ cells. **G** m6A enrichment of ZEB1 in MCF-7^SN-exo^ and MDA-MB-231^SN-exo^ cells with or without FTO silencing. **H** Luciferase reporter assays showing the effect of FTO on ZEB1 reporters with wild-type (WT) or m6A mutated binding sites. **I** The indicated cells were transiently transfected with shFTO or shNC plasmids. The half-life (t1/2) of ZEB1 mRNA was examined. **J** CLIP-qPCR showing the association of ENO1 transcripts with WTAP in MCF-7^SN-exo^ and MDA-MB-231^SN-exo^ cells. **K** qPCR analysis of ZEB1 expression in tumor cells with and without FTO depletion followed by indicated exosomes treatment for 48 h. Data represent the mean ± SD of at least three independent experiments. *P < 0.05, **P < 0.01, ***P < 0.001, ns, not significant.

It is observed that piRNA-17560 has one binding site within the 3′UTR of FTO (Fig. 5A). To verify that FTO was a direct target of piR-17560, a dual luciferase reporter assay was performed. As shown in Fig. 5B, piR-17560 silencing caused a significant decrease in the luciferase activity of the reporter construct carrying the wild-type FTO 3′UTR relative to the control. This decrease was dramatically abrogated when the putative binding site was mutated (Fig. 5B). Moreover, piRNA-17560 knockdown diminished the FTO mRNA stability in both MCF-7^SN-exo^ and MDA-MB-231^SN-exo^ cells, as we measured the half-life of FTO mRNA by inhibition of transcription with actinomycin D (Fig. 5C). Conversely, ectopic overexpression of piRNA-17560 enhanced the stability of FTO mRNA (Fig. 5D), as well as its protein expression in MCF-7 and MDA-MB-231 cells (Fig. 5E). Therefore, piRNA-17560 alleviates the decay of FTO mRNA and enhances its stability by binding to the 3’UTR of FTO.

Fat mass and obesity-associated protein (FTO) is one of key m6A mRNA demethylases. Here, we investigated whether exosomal piRNA-17560-induced FTO could regulate m6A methylation to promote ZEB1 expression. First, we found that the global m6A level in MCF-7^SN-exo^ and MDA-MB-231^SN-exo^ cells was diminished compared with that in the control cells, which was increased by FTO depletion (Supplementary Fig. 6A). The existence of m6A modification on ZEB1 was then examined by m6A-RIP sequencing. Through analyzing the sequencing profiles, we detected several m6A peaks that were upregulated by FTO silencing in 3′UTR of the ZEB1 mRNA (Fig. 5F). Indeed, knockdown of FTO enriched m6A-methylated ZEB1 mRNAs in MCF-7^SN-exo^ and MDA-MB-231^SN-exo^, which was consistent with the m6A-dependent function of FTO (Fig. 5G). We then constructed luciferase reporters containing 3′UTR of ZEB1 (pGL3-ZEB1-WT) or control mutant (pGL3-ZEB1-MUT). As expected, silencing FTO alleviated the luciferase activity of the pGL3-ZEB1-WT vector, while mutation in the m6A sites abolished this effect (Fig. 5H). In addition, knockdown of FTO decreased ZEB1 mRNA stability in both MCF-7^SN-exo^ and MDA-MB-231^SN-exo^ cells (Fig. 5I). To confirm the direct interaction between ZEB1 mRNAs and FTO protein, we further performed cross-linking and RNA immunoprecipitation (CLIP) qPCR assay. As shown in Fig. 5J, ZEB1 mRNAs could interact with FTO in MCF-7^SN-exo^ and MDA-MB-231^SN-exo^, suggesting that ZEB1 is direct target of FTO. Moreover, FTO silencing reduced mRNA levels of ZEB1 in SN-exo-cultured tumor cells (Fig. 5K). Based on these observations, we demonstrate that exosomal piR-17560 from senescent neutrophils upregulates ZEB1 expression via FTO-mediated m6A demethylation.

### YTHDF2 is essential for the posttranscriptional regulation of ZEB1

**Fig. 6:**
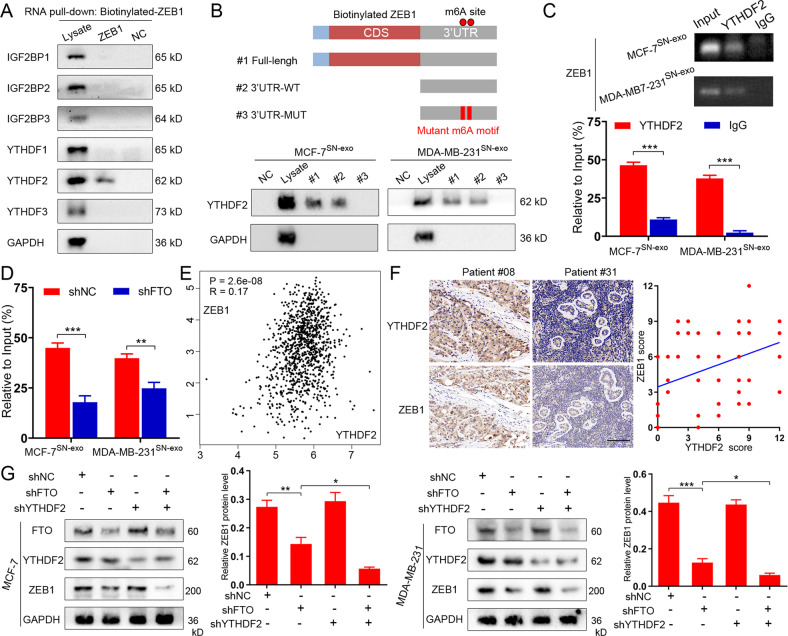
YTHDF2 is essential for the posttranscriptional regulation of ZEB1 by piR-17550/FTO signaling. **A** RNA pulldown was performed with biotinylated ZEB1. Immunoblotting of IGF2BP family and YTH family m6A readers in cell lysate, biotinylated full-length ZEB1 and beads only (NC) in MCF-7^SN-exo^ cells. **B** Immunoblotting of ZEB1 full length (#1), ZEB1 3’UTR region (#2), 3’UTR m6A motif mutant ZEB1 (#3), lysate (Ly) and beads only (NC) by RNA pulldown assay in MCF-7^SN-exo^ and MDA-MB-231^SN-exo^ cells. **C** RIP assays showing the direct binding between YTHDF2 protein and ZEB1 mRNA in HCT116 and DLD1 cells. Agarose electrophoresis (up) and qPCR analysis (down). **D** RIP assays demonstrating the enrichment of YTHDF2 protein bound ZEB1 mRNA in shNC versus shFTO MCF-7^SN-exo^ and MDA-MB-231^SN-exo^ cells. **E** The correlation between YTHDF2 and ZEB1 in breast cancer was analyzed in TCGA. **F** Expression correlation of YTHDF2 and ZEB1 was analyzed in breast cancer patients (*n* = 82) using IHC. Scare bars, 100 μm. **G** Immunoblotting of YTHDF2, FTO and ZEB1 protein levels in MCF-7^SN-exo^ and MDA-MB-231^SN-exo^ cells with shNC, FTO knockdown only (shFTO#1), YTHDF2 knockdown only (shYTHDF2) and both FTO and YTHDF2 knockdown (shFTO + shYTHDF2). Data represent the mean ± SD of at least three independent experiments. *P < 0.05, **P < 0.01, ***P < 0.001, ns, not significant.

“Writers” and “erasers” determine m6A prevalence and distribution, whereas “readers” regulate the downstream effects. To date, several m6A reader proteins have been identified, including members of the insulin-like growth factor 2 mRNA-binding protein (IGF2BP) family and the YT521-B homology (YTH) family [14]. RNA pulldown assay was used to identify the specific m6A readers of ZEB1 transcripts. We found that YTHDF2, but not other readers, specifically bound to the ZEB1 transcripts in MCF-7^SN-exo^ cells (Fig. 6A). The direct binding of YTHDF2 and full-length transcripts of ZEB1 were also verified in both MCF-7^SN-exo^ and MDA-MB-231^SN-exo^ cells, and the specific binding was found to be significantly impaired after m6A motif mutation (Fig. 6B and Table S2). In addition, the RIP assay verified the direct binding of YTHDF2 protein and ZEB1 mRNA in MCF-7^SN-exo^ and MDA-MB-231^SN-exo^ cells (Fig. 6C), and the interaction between YTHDF2 and ZEB1 was alleviated after FTO knockdown (Fig. 6D). Clinically, YTHDF2 expression exhibited a positive correlation with ZEB1 expression in both TCGA database (Fig. 6E) and BC tumor tissues (*n* = 82) from our independent cohort (Fig. 6F). The depletion of FTO downregulated ZEB1 expression, while this effect was further enhanced by simultaneous YTHDF2 knockdown in MCF-7 and MDA-MB-231 cells (Fig. 6G). Taken together, the RNA-binding protein YTHDF2 functions collectively with FTO to affect ZEB1 mRNA stability and expression in BC cells.

### Senescent neutrophils produce exosomal piR-17560 in a STAT3-dependent manner

**Fig. 7:**
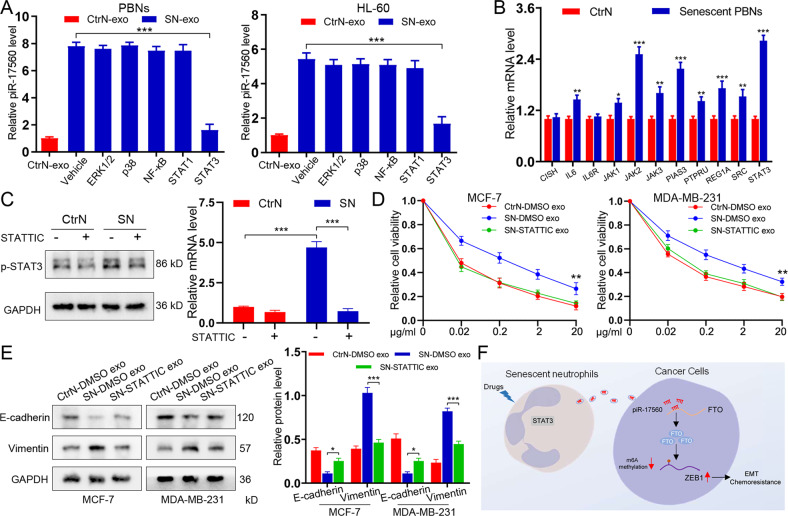
Senescent neutrophils produce exosomal piR-17560 in a STAT3-dependent manner. **A** qPCR analysis of piR-17560 expression in PBNs and HL-60 cells treated with inhibitors of ERK1/2 (SCH772984), p38 (SB203580), NF-κB (JSH-23), STAT1 (Fludarabine) and STAT3 (STATTIC) for 12 h. **B** qPCR analysis showing the enrichment of STAT3 activation signature in senescent neutrophils relative to controls. **C** Senescent or control neutrophils were treated with STATTIC (1 µM) for 24 h, and examined for p-STAT3 expression by immunoblotting. **D** CCK8 assay of MCF-7 and MDA-MB-231 cells pre-incubated with indicated exosomes for 48 h followed by docetaxel at different concentrations for 48 h. **E** Immunoblotting of E-cadherin and Vimentin in tumor cells re-incubated with indicated exosomes for 48 h. **F** Schematic model for senescent neutrophils in promoting chemoresistance and EMT of breast cancer cells via exosomal piR-17560. Data represent the mean ± SD of at least three independent experiments. *P < 0.05, **P < 0.01, ***P < 0.001.

We next sought to explore the mechanism supporting the increased secretion of exosomal piR-17560 from senescent neutrophils. It has been documented that several signaling signatures are activated in senescent cells [15]. To this end, we screened these potential pathways by using inhibitors that specifically targeting ERK1/2, p38, NF-κB, STAT1, and STAT3, respectively. We found the kinase inhibitor for STAT3 efficiently suppressed the generation of exosomal piR-17560 in senescent PBNs in a 24 h period (Fig. 7A). Indeed, STAT3 activation signature was significantly enriched in senescent neutrophils relative to controls (Fig. 7B). Meanwhile, we found a significant upregulation of p-STAT3 in senescent neutrophils, this effect was largely abolished in the presence of STATTIC (Fig. 7C). Interestingly, the number of exosomes was not diminished in the cells that were cultured with STATTIC at the same dose (Supplementary Fig. 6B). In addition, the STATTIC treatment significantly abated chemoresistance (Fig. 7D) and EMT (Fig. 7E) of cancer cells induced by SN-exo. To further validate this result, we treated PBNs with STAT3-specific siRNA in the presence or absence of SN-exo. We found that STAT3 knockdown led to a significant decrease in the exosomal production of piR-17560 (Supplementary Fig. 6C). Moreover, the introduction of STAT3 siRNAs abolished the SN-exo-mediated docetaxel resistance (Supplementary Fig. 6D) and EMT (Supplementary Fig. 6E). Altogether, senescent neutrophils produce exosomal piR-17560 in a STAT3-dependent manner.

Collectively, the diagram of this study is present in Fig. 7F.

## Discussion

Mutual interaction between cancer cells and the surrounding environment is critical for tumor development. Exosomes have recently emerged as a novel mechanism to modulate cell-cell contacts, which can be secreted by various cell types [16]. Exosomes contain a range of proteins, lipids, RNAs and DNA fragments. To date, most cancer-related exosomes studies focus on cancer cells [17]. On the contrary, here, we present evidence to indicate that senescent neutrophils, which abundantly reside in chemotherapy-treated breast cancer tissues, are responsible for tumor progression by producing exosomes to disseminate drug resistance.

piRNAs are small noncoding RNAs consisting of 24-32 nucleotides that specifically bind to PIWI proteins [18, 19]. Despite the usual knowledge that piRNAs have significant functions in germline development, a growing body of evidence reveals that piRNAs also play roles in tumorigenesis and are relevant to the prognosis of cancer [20]. It is reported that piRNA-36712 restrains breast cancer progression and chemoresistance [21]. Conversely, piRNA-823 is described to promote luminal breast cancer development by inducing cancer stemness [22]. Recently, several studies highlighted the role of piRNAs in exosomes. The piRNAs from exosomes may participate in biological regulation, antiviral immunity, and growth of neighboring cells [23–26]. However, few studies have explored the function of exosomal piRNAs in malignant development.

In this study, by performing small RNA-seq of exosomes, we found that the exosomal piR-17560 was highly generated by senescent neutrophils in a STAT3-dependent manner. Moreover, the exosomal piR-17560 could sufficiently confer docetaxel tolerance to BC cells. When overexpressing piR-17560 or electroporating the PBNs-derived exosomes with piR-17560, BC cells were also endowed with drug-resistant property. In addition, the clinical significance of exosomal piR-17560 was highlighted in breast cancer samples. However, due to the difficulty in extracting exosomes from TINs, PBNs were used in most of experiments. This limitation must be acknowledged about our exploration.

EMT, a pivotal biological phenomenon involved in embryonic development, has been characterized to modulate tumor invasion and metastasis [27]. EMT could also allow cancer cells to acquire self-renewal capability, as well as tolerance traits to chemotherapy [28]. Previous exploration has suggested the enrichment of cells expressing mesenchymal markers in breast cancer upon treatments [29]. Interestingly, although EMT is linked to the escape from cellular senescence during tumor initiation [30], our study shows that senescent neutrophils can secret exosomes to induce EMT-related chemoresistance of BC cells, and this effect is dependent on ZEB1. ZEB1 is a prime transcription factor that initiates EMT in cancer cells. It has been reported that ZEB1 is regulated by various signals at both transcriptional and post-transcriptional level, like signal transducer and activator of transcription 3, insulin-like growth factor 1 and noncoding RNAs [31–33]. In this work, we identified a new epigenetic regulatory mode of ZEB1 expression, which relied on FTO-mediated m6A modification.

m6A is the most common internal modification in eukaryotic mRNA and mediates the stability and translation efficiency of mRNAs [34]. m6A plays a crucial role in carcinogenesis through the m6A enzyme system, which is composed of RNA-binding proteins, methyltransferases, and demethylases [35]. There are two known m6A demethylases, AlkB homolog 5 and FTO. FTO was initially described as a gene linked to obesity and energy homeostasis and was then recognized as the nucleic acid demethylase [36]. The function of FTO is inconsistent among different types of malignancies. FTO was observed to play tumor-suppressor roles in ovarian cancer and hepatocellular carcinoma [37, 38]. Meanwhile, some studies demonstrated that FTO had oncogenic roles in breast cancer, acute myeloid myeloid leukemia and melanoma [39–41]. Indeed, our investigation confirmed the significance of FTO-mediated m6A demethylation in BC development. Moreover, this effect is dependent on the RNA-binding protein YTHDF2.

Like other noncoding RNAs that can induce mRNAs degradation, piRNAs are also capable of forming a silencing complex to facilitate mRNAs decay. It has been documented that piRNAs prefer to modulate transposable elements’ transcription, resulting in the downregulation of target genes at post-transcriptional level [18]. However, in this study, we demonstrated that the exosomal piRNA-17560 increased, but not decreased FTO expression level in BC cells. Mechanically, piRNA-17560 achieved this effect by targeting a complementary sequence in the 3’UTR of FTO to enhance its stability. Of note, our findings are consistent with a previous study, which also shows that piRNA upregulates the expression of downstream gene [42]. Thus, the function of piRNAs in gene modification needs further exploration.

Overall, we demonstrate that exosomes from senescent neutrophils could endow recipient BC cells with chemoresistance and EMT characteristics via intercellular transfer of piR-17560. Moreover, the exosomal piR-17560 promotes ZEB1-induced EMT of BC cells by FTO-dependent m6A demethylation. These findings reveal the critical role of senescent neutrophils in regulating EMT and provide a therapeutic target for treatment resistance in breast cancer patients.

## Materials and methods

### Clinical samples and neutrophil isolation

Tumor tissues and peripheral blood were taken from BC patients who underwent surgery at the First Hospital of Anhui Medical University between 2020 and 2021. Written informed consent was obtained from each patient. The isolation of neutrophils was conducted as previously described [9]. Briefly, fresh tumor tissues were cut into pieces and digested in RPMI-1640 with 20% fetal bovine serum, 0.002% DNase I (Roche), and 0.05% collagenase IV (Sigma-Aldrich) at 37 °C for 30 min. The cells suspension was then filtered through a 70-μm mesh and stained with antihuman CD66b antibody. We sorted the neutrophils by using fluorescence-activating cell sorter (BD Biosciences). For the collection of PBNs, fresh whole blood of patients or healthy donors was mixed with an equal volume of HBSS and was layered on top of Ficoll-Hypaque 1077 (Sigma-Aldrich) in 1:1 ratio and centrifuged. The PBNs were isolated by staining CD66b with FACS. The sorted cells were not used unless a purity of >85%. This study was performed under the Ethics Committee of the First Hospital of Anhui Medical University approval.

### Cells and reagents

BC cell lines (MCF-7, MDA-MB-231) and human neutrophils (HL-60) were purchased from the Chinese Academy of Sciences and grown in either RPMI-1640 or DMEM. All cells were authenticated by short tandem repeat profiling and tested for mycoplasma. To target specific pathways, neutrophils were treated with vehicle (DMSO), 10 μM SCH772984, 6 mM JSH-23, 5 μM SB203580, 50 μM Fludarabine or 5 μM STATTIC for 12 h prior to the experiments. These inhibitors were purchased from Selleckchem (USA).

### Exosome experiments

Cell culture medium was filtered through a 0.22 μm PVDF filter (Millipore, USA). We then collected exosomes using standard extraction methods as previously described [43]. The number and size of exosomes were examined by Electron Microscopy and quantified by NanoSight NS300 instrument (UK). For RNA and protein isolation, exosomes were first cultured with RNase or Proteinase K, respectively. Equal number of exosomes were used for qPCR with exogenous λ polyA as the control; equal number of exosomes used for protein experiments were suspended in SDS lysis buffer.

For in vitro tests, 1 µg exosomes were incubated with 2 × 10^5^ recipient cells. Electroporation of piRNA into exosomes was performed using GenePulser XcellTM electroporation system (BioRad, USA) according to the protocols. Briefly, 400 nM RNA and 2 µg exosomes were mixed in 400 µl electroporation buffer and electroporated at 350 V and 150 µF. The mixture was then hatched at 37 °C for 30 min to make the membrane of exosomes fully recovered, following by RNase treatment.

### Fluorescence in situ hybridization (FISH) and immunofluorescence

Fluorescence-conjugated piRNA-17560 probe for RNA FISH were generated by GenePharma (China). Cells were treated by 10% paraformaldehyde to be fixed, following by hybridization with RNA probe. Nuclei were counterstained with DAPI. All the experiments were conducted according to the FISH protocols. For immunofluorescence, cells were fixed by 10% paraformaldehyde, permeabilized by 0.1% Triton-100 and blocked by donkey serum. Cells were then incubated with the primary antibody (Vimentin, 1:500, Abcam) overnight at 4 °C, followed by Alexa-594-conjugated secondary antibody (1:1000, Abcam). The samples were visualized by fluorescence microscope (Olympus, Japan).

### Immunoblotting

Immunoblotting was carried out as previously described [44]. Antibodies against E-cadherin (3195, 1:1000), Vimentin (5741, 1:1000), p-STAT3 (9145, 1:1000), ZEB1 (70512, 1:1000) and β-actin (4970, 1:1000) were purchased from Cell Signaling Technology. Antibodies against FTO (ab126605, 1:1000), YTHDF1 (ab252563, 1:1000), YTHDF2 (ab246514, 1:1000), YTHDF3 (ab220161, 1:1000), TSG101 (ab125011, 1:1000), and CD81 (ab134045, 1:1000) were purchased from Abcam. Antibodies against IGF2BP1 (22803-1-AP, 1:1000), IGF2BP2 (11601-1-AP, 1:1000), IGF2BP3 (14642-1-AP, 1:1000) and GAPDH (60004-1-Ig, 1:2000) were purchased from Proteintech (China). Full and uncropped blots were uploaded as Supplemental Material.

### Quantitative real-time PCR (qPCR), RNA-seq, and RNA decay assays

Total RNA was extracted with TRIzol (Invitrogen) and cDNA was synthesized by PrimeScript RT Master Mix (Takara). RNA levels were determined with ABI 7900HT Real-Time PCR system using the SYBR Green method. piRNA quantification was determined by using Bulge-loop^TM^ miRNA qRT-PCR Primer Set (RiboBio, China). The RNA level was normalized against GAPDH RNA, and the piRNA levels were normalized against U6. The primer sequences are shown in Tables S3–4. Small RNA-seq was conducted as a service at Majorbio (Shanghai, China) and sequenced on HiSeq1500 (Illumina) for 50 cycles. The piRNA sequences were annotated according to piRBase (http://bigdata.ibp.ac.cn/piRBase). Raw data of the exosomes sequencing are available in The Sequence Read Archive with the accession number PRJNA830989. Gene Set Enrichment Analysis (GSEA) was performed to explore the potential signaling pathways. For RNA decay assays, cells were treated with actinomycin D (Santa Cruz, 5 µM) and collected at the indicated time for qPCR analysis. The half-life (t1/2) of mRNA was measured and normalized to GAPDH.

### Immunohistochemistry (IHC)

Clinical specimens embedded with paraffin were made into tissue microarray. The staining of tissue was conducted as previously documented [45]. The results of each case were determined by the multiplication of two scores, referring to staining intensity and the percentage of positive cells (staining intensity score: negative, 0; weak, 1, moderate, 2; strong, 3; and the percentage score: 0, 0–5%; 1, 6–10%; 2, 11–30%; 3, 31–60%; 4, 61–100%). Immunohistochemical score was independently evaluated by two explorers who were blinded to clinical information.

### piRNA-17560 silencing by antagomir

It has been shown that cholesterol and 2′-O-methylation modifications were effective in delivering antagomirs-mediated silencing effects [46, 47]. Here, the chemically modified hsa-piRNA-17560 antagomir (antimir17560) was used to inhibit piRNA-17560 expression, and nontarget antagomir (antimirNC) was used as a negative control. The antimir17560 sequence is 5′-AAG GGA CCC TCC CTA TCA CCA CCT TCT TTA-3′. The sequence of antimirNC is 5′-UUG UAC UAC ACA AAA GUA CUG-3′. We carried out 2′-O-methylation modification in all bases, and added cholesterol to the 3′ end of antagomirs. Transfection of the antagomirs was performed using Lipofectamine 3000 (Invitrogen) according to manufacturer’s recommendation.

### Cell transfection

The lentivirus carrying shRNA constructs and siRNA oligonucleotides were provided by Genepharma (Shanghai, China). The shRNA targeting sequences and siRNA were listed in Table S5. Lentivirus particles were transfected into tumor cells in the presence of polybrene (8 µg/mL) and selected by using 5 μg/ml puromycin. All transfected cells were tested regularly by immunoblotting to ensure the interfering efficiency. For the transfection of siRNAs, Lipofectamine 3000 (Invitrogen) was used in accordance with the instructions.

### CCK-8, EdU, and migration assay

The viability of cells was determined by Cell Counting Kit 8 (Dojindo, Japan) and measured at OD450 nm with the BioTek Gen5 system (BioTek, USA). 5-ethynyl-2′-deoxyuridine (EdU) was performed to evaluate cells proliferation according to the protocols of Click-iT EdU Assay (Roche, USA). Migration assays were performed using a Transwell system (Corning, USA). A total of 1 × 10^5^ cells in 200 µl serum-free medium were seeded in upper chamber. The lower chamber was cultured with 600 µl of medium containing 20% FBS. After 24 h, cells were fixed with methanol and stained with crystal violet. The number of migrated cells was then counted.

### Xenograft model

The animal experiments were conducted with the approval of the Animal Ethics Committee (Anhui Medical University). Four-week-old female athymic nude mice were used (seven per group) and housed in a standard pathogen-free conditions. A total of 1 × 10^6^ luciferase-labeled tumor cells were injected subcutaneously with SN-exo or Ctr-exo. At day 7 after inoculation (the tumors reached ~100 mm^3^), exosomes were injected intratumorally every third day (5 μg per time), followed by intraperitoneal administration of docetaxel (10 mg/kg, once per week). Tumor size was measured every week and calculated using the formula: *V* = (Width^2^ × Length)/2. After 6 weeks, mice were imaged by bioluminescent imaging technology (IVIS Illumina System) and the xenografts were harvested for subsequent analysis.

### Luciferase reporter assay

The miRanda algorithm (www.microrna.org) was used to identify the putative binding site of piRNA-17560 at the FTO 3′UTR. We amplified the wild-type and mutant target sites at the FTO 3′UTR and then cloned them into a pmirGLO vector. Furthermore, to examine whether FTO might affect ZEB1 expression, the 3′UTR of ZEB1, which contained m6A methylation sites, were cloned into a pGL3-basic vector and then cotransfected with a pRL-CMV plasmid containing the renilla luciferase gene. The tumor cells were seeded in 24-well plates and cotransfected with both the wild-type or mutant constructs with either FTO knockdown or control using Lipofectamine 3000 (Invitrogen). Cells were harvested at 48 h, the luciferase activity was detected using the Dual-Luciferase Reporter Assay System (Promega, USA). Primers for construction of luciferase reporter plasmids are listed in Table S6. The specific sequences of wild-type or m6A motif mutant ZEB1 3′UTR are shown in Table S2.

### m6A Quantification

We first purified the mRNA by using two rounds of the Dynabeads mRNA Purification Kit (Thermo Scientific). Then, the change in the global m6A levels in mRNA was tested by an EpiQuik m6A RNA Methylation Quantification Kit (Epigentek, Germany) following the manufacturer’s protocol. Poly-A-purified RNA (200 ng) was used for sample analysis.

### m6A RNA immunoprecipitation (m6A-RIP) qPCR and sequencing

m6A-RIP qPCR and sequencing were performed as previously documented [48]. Total RNA was first extracted by from tumor cells and sheared into ~100-nt-long fragments with RNA fragmentation reagents (Invitrogen). Fragmented RNA was mixed with anti-m6A antibody (ab208577, Abcam) for 1 h at 4 °C. Then, the fragmented RNA and m6A antibody mixture were conjugated to beads by rotation overnight. RIP was performed according to the instructions of Magna Methylated RNA Immune-precipitation m6A Kit (Millipore, USA). We then purified the methylated RNA and examined its expression by qPCR with the primers shown in Table S3. In addition, 100 ng purified RNA from m6A-RIP were used for library building with NEBNext Ultra RNA Library Prep Kit for Illumina (NEB, USA). Sequencing was carried out on Illumina HiSeq 2500 according to the manufacturer’s protocol. The primary data are deposited in The Sequence Read Archive with the accession number PRJNA830990. Effective reads from sequencing were aligned to the human genome (GRCh38/hg38), and m6A peak calling was performed by magnetic cell sorting as described.

### Cross-linking and RNA immunoprecipitation (CLIP-qPCR)

The interaction between FTO and ZEB1 mRNA was validated by CLIP-qPCR. Cells at 80% confluence were cross-linked by UV and harvested. The nuclear fraction was then collected and lysed in RIP buffer with RNAase inhibitors and protease inhibitors for sonication. For each reaction, 1 mg of FTO antibody or control IgG antibody were conjugated to Protein A/G magnetic beads (Santa Cruz) by incubation for 4 h at 4 °C. The conjugated beads were washed with RIP buffer, incubated with prepared nuclear contents at 4 °C overnight. After three times washing, the beads were incubated with RNase-free DNase I and Proteinase K for 15 min at 37 °C, respectively. The input and immunoprecipitated RNAs were recovered by TRIzol extraction and used for subsequent qPCR analyses.

### m6A mutation assays

SRAMP (http://www.cuilab.cn/sramp/) was used to predict potential m6A modification sites on ZEB1 mRNA sequences. Full-length ZEB1 transcripts, the ZEB1 untranslated region (3′UTR) and the m6A motif mutant 3′UTR regions were cloned into pGL3 plasmids and used for the RNA pulldown assay.

### RNA pull-down

Biotin-labeled RNA was in vitro transcribed using MEGAscript T7 Transcription Kit (Thermo Scientific). The Pierce Magnetic RNA-Protein Pulldown Kit (Thermo Scientific) was utilized according to the manufacturer’s protocol. Labeled RNA was first incubated with cytoplasmic extract of BC cells at room temperature for 1 h. Then, the prewashed streptavidin beads were added to each binding reaction and further cultured for 1 h. Precipitates were washed for five times and boiled with SDS buffer for 10 min followed by immunoblotting analysis.

### RNA immunoprecipitation (RIP)

RIP assay was performed by the Magna RIPTM RNA-Binding Protein Immunoprecipitation Kit (Millipore, USA). In brief, the harvested tumor cells were incubated with magnetic beads that coated with anti-YTHDF2 antibody (ab246514, Abcam) or isotype IgG at 4 °C overnight. Then the beads were washed six times and incubated with proteinase K for digestion. We used Phenol-chloroform-isoamyl alcohol reagent to extract the RNA in the precipitates and inputs. Reverse transcription PCR was conducted to quantify ZEB1 mRNA and the expression was normalized to control input. IgG was used to confirm the specificity of RNA–protein interactions.

### Statistical analysis

All data are presented as the means ± SD. The experiments were repeated independently at least three times. Unpaired two-sided Student’s *t*-test and one-way analysis of variance were used for the data analysis. Pearson correlation analysis was used to determine the correlation between two variables. Patients’ survival outcome was assessed by Kaplan–Meier method. A *p*-value lower than 0.05 was considered significant.

## Supplementary information

Supplementary Figure Legends

Supplementary Figure 1

Supplementary Figure 2

Supplementary Figure 3

Supplementary Figure 4

Supplementary Figure 5

Supplementary Figure 6

Supplementary Table

Original Data File

Original Data File

Reproducibility Checklist

Author Contribution Form

## Data Availability

The dataset supporting the conclusions of this article is included within the article. The sequencing data are deposited in The Sequence Read Archive with the accession number PRJNA830989 and PRJNA830990.
