# Supplementary material for: Senescent neutrophils-derived exosomal piRNA-17560 promotes chemoresistance and EMT of breast cancer via FTO-mediated m6A demethylation
Source: Cell Death Dis. 2022 Oct 27;13(10):905. doi: 10.1038/s41419-022-05317-3 (PMC9613690; doi:10.1038/s41419-022-05317-3)
Supplement: Supplementary file 1 — Supplementary Figure Legends [file 41419_2022_5317_MOESM1_ESM.docx]

**Supplementary Fig. 1** (A) Kaplan–Meier survival analysis showing overall survival based on the expression of senescent neutrophil gene signature in GSE65194. (B) Quantification of SA-βGal staining in PBNs and HL-60 with and without doxorubicin (DXR) treatment. (C) DNA damage repair (DDR) profiling of PBNs and HL-60 upon immunofluorescence staining. Cells were divided into 4 subgroups according to the number of DDR foci. (D) qPCR analysis of SASP factors (CSF3, CCL3, CXCL8 and IL1α) in PBNs and HL-60 with and without doxorubicin (DXR) treatment. (E) CCK8 assay of tumor cells pre-incubated with indicated exosomes for 48 h followed by doxorubicin (DXR) treatment at indicated concentrations for 48 h. (F) qPCR analysis showing the interfering efficiency of RAB27A and RAB27B siRNA in PBNs. (G) CCK8 assay of PBNs transfected with RAB27A/B siRNA or treated with GW4869 (5 μM) at indicated time. *P < 0.05, ***P < 0.001.

**Supplementary Fig. 2** (A) The viability of cancer cells transfected with piR-805 or piR-17033 mimics followed by docetaxel at different concentrations for 48 h. (B) qPCR analysis of piR-17560 expression in MCF-7 and MDA-MB-231 treated with Actinomycin D (1 μg/mL) followed by indicated exosomes treatment for 48 h. (C) qPCR analysis of piR-17560 expression in tumor cells incubated with the piR-17560-electroporated exosomes. (D) qPCR analysis of piR-17560 expression in the exosomes produced from control and senescent TINs. (E) CCK8 assay of tumor cells pre-incubated with indicated exosomes for 48 h followed by docetaxel treatment at indicated concentrations for 48 h. ***P < 0.01, ***P < 0.001, ns, not significant.

**Supplementary Fig. 3** (A) qPCR analysis of exosomal piR-17560 expression in healthy donors and breast cancer patients with and without chemotherapy. (B) qPCR analysis of exosomal piR-17560 expression in the plasma of patients before and after chemotherapy. (C) qPCR analysis of exosomal piR-17560 expression in post-therapy plasma in BC patients with SD+PD (n = 33) or CR+PR (n = 38) during DTX-based neoadjuvant therapy. *P < 0.05, ***P < 0.001.

**Supplementary Fig. 4** (A) Immunoblotting of E-cadherin, Vimentin, ZEB1 and FTO in BC cells incubated with indicated exosomes. Densitometry represents the expression of the proteins relative to GAPDH. (B) The morphology of MCF-7 and MDA-MB-231 cells treated with indicated exosomes for 48 h. Scare bars, 50 μm. (C) Immunofluorescence staining of Vimentin in MCF-7 and MDA-MB-231 cells treated with indicated exosomes. Scare bars, 50 μm. (D) qPCR analysis of FTO expression in tumor cells incubated with the exosomes from piRNA-17560-depleted senescent neutrophils. (E) Immunoblotting of FTO, E-cadherin, Vimentin and ZEB1 in BC cells with and without FTO knockdown upon indicated exosomes treatment. Densitometry represents the expression of the proteins relative to GAPDH. *P < 0.05, **P < 0.01, ***P < 0.001.

**Supplementary Fig. 5** (A) The morphology of MCF-7 and MDA-MB-231 cells treated with indicated exosomes for 48 h. Scare bars, 50 μm. (B) Immunoblotting of ZEB1 and FTO in tumor cells with indicated exosomes treatment. Densitometry represents the expression of the proteins relative to GAPDH. (C) IHC showing a high expression of FTO and ZEB1 in SN-exo-treated xenografts than CtrN-exo-treated xenografts. (D) IHC showing a higher expression of FTO and ZEB1 in patients who suffered from SD or PD than those with CR or PR during DTX-based therapy. (E) The correlation between FTO and ZEB1 in human breast cancer was analyzed in TCGA. ***P < 0.001.

**Supplementary Fig. 6** (A) MCF-7 and MDA-MB-231 cells with or without FTO inhibition were treated with the indicated exosomes and tested by the EpiQuik™ m6A RNA methylation quantification kit. (B) Quantification of the number of exosomes from senescent or control neutrophils with or without STATTIC treatment. (C) qPCR analysis of piR-17560 expression in senescent or control neutrophils derived exosomes. (D) CCK8 assay of MCF-7 and MDA-MB-231 cells pre-incubated with indicated exosomes for 48 h followed by docetaxel at different concentrations for 48 h. (E) Immunoblotting of E-cadherin and Vimentin in MCF-7 and MDA-MB-231 cells incubated with indicated exosomes. Densitometry represents the expression of the proteins relative to GAPDH. *P < 0.05, **P < 0.01, ***P < 0.001. ns, not significant.
