# Supplementary figures and images for: Senescent neutrophils-derived exosomal piRNA-17560 promotes chemoresistance and EMT of breast cancer via FTO-mediated m6A demethylation
Source: Cell Death Dis. 2022 Oct 27;13(10):905. doi: 10.1038/s41419-022-05317-3 (PMC9613690; doi:10.1038/s41419-022-05317-3)

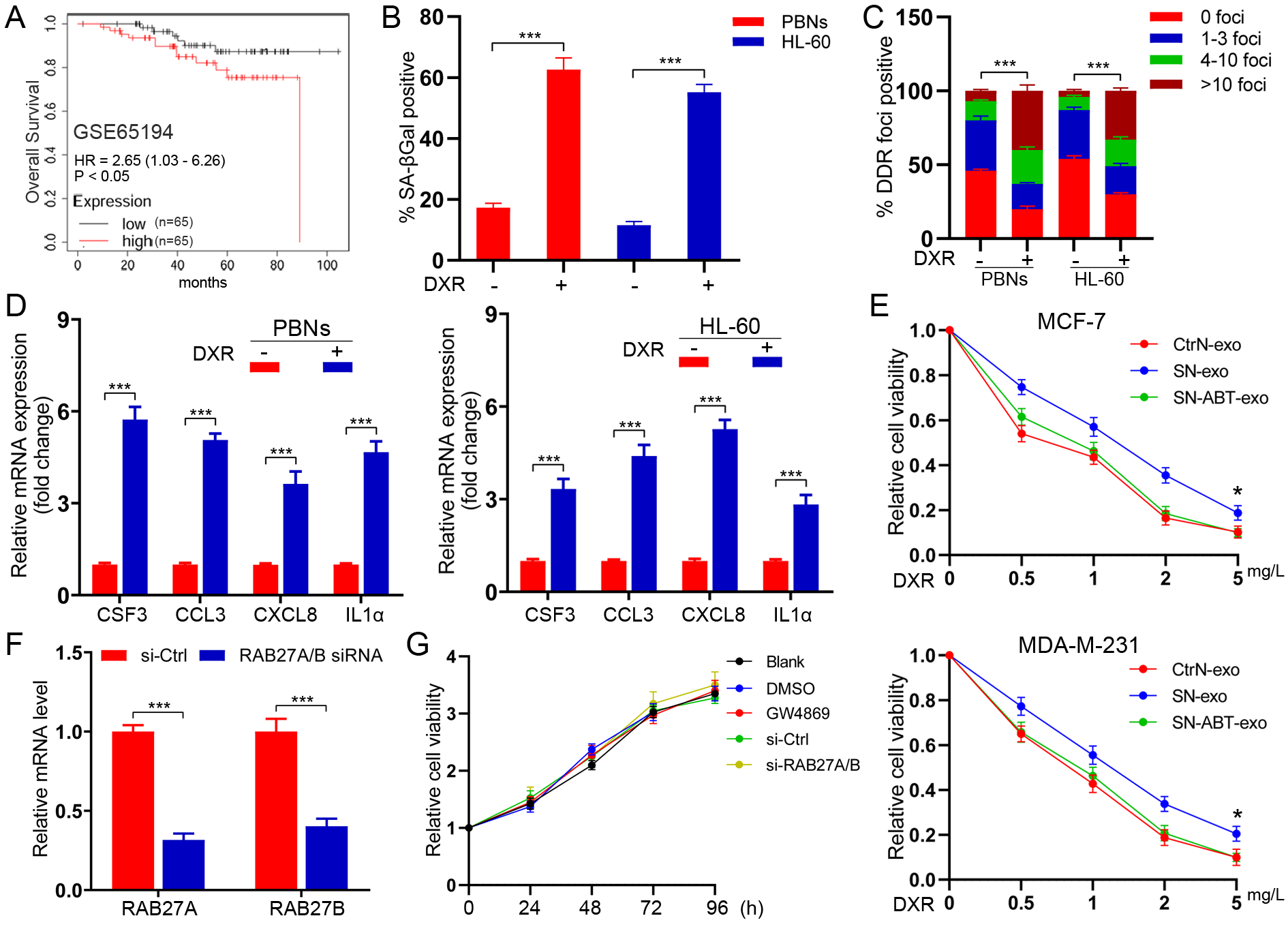

Supplement: Supplementary file 2 — Supplementary Figure 1 [file 41419_2022_5317_MOESM2_ESM.tif]

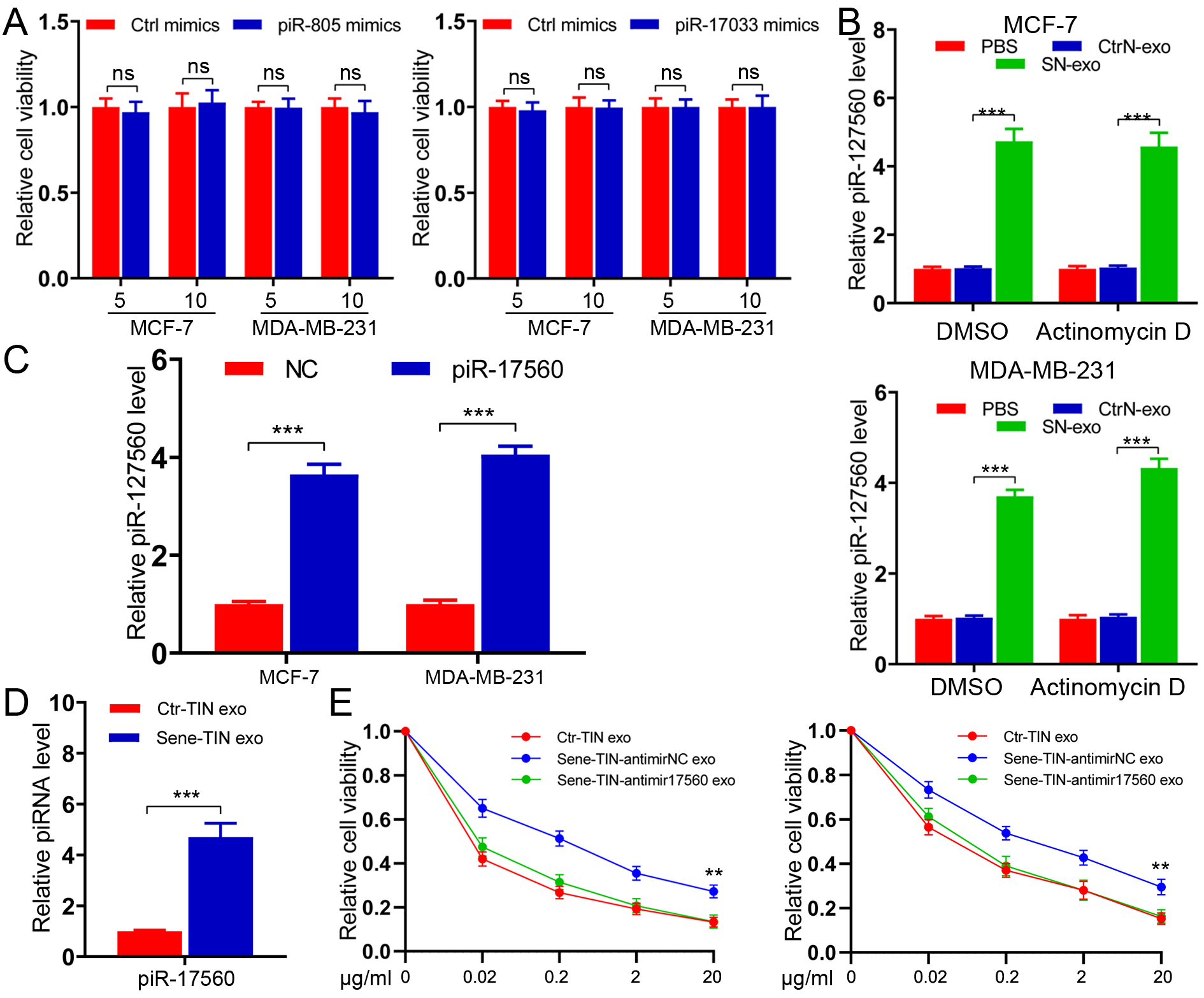

Supplement: Supplementary file 3 — Supplementary Figure 2 [file 41419_2022_5317_MOESM3_ESM.tif]

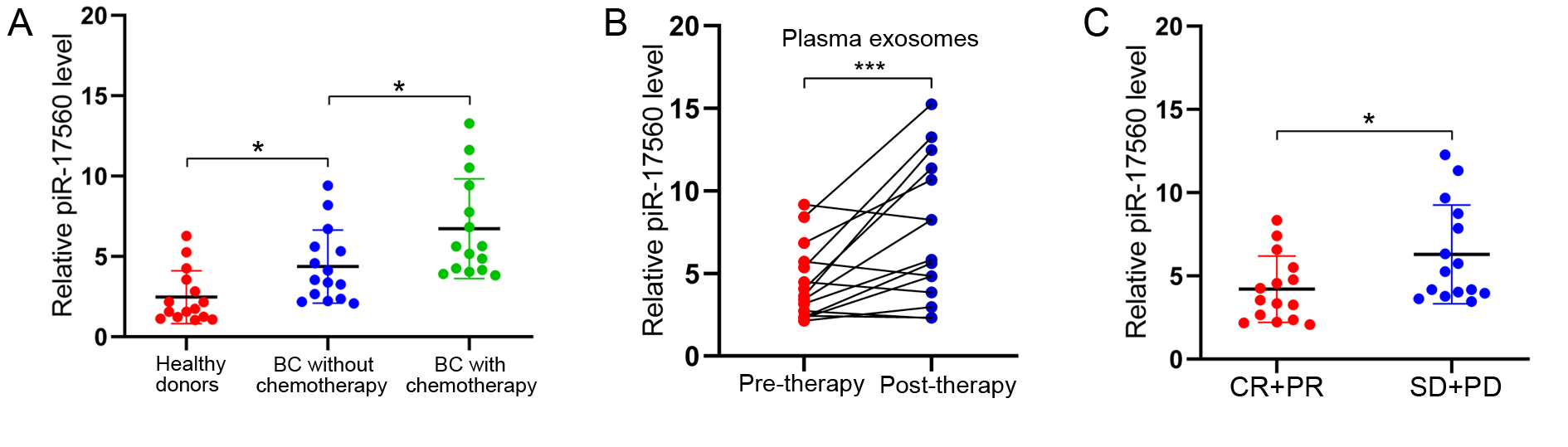

Supplement: Supplementary file 4 — Supplementary Figure 3 [file 41419_2022_5317_MOESM4_ESM.tif]

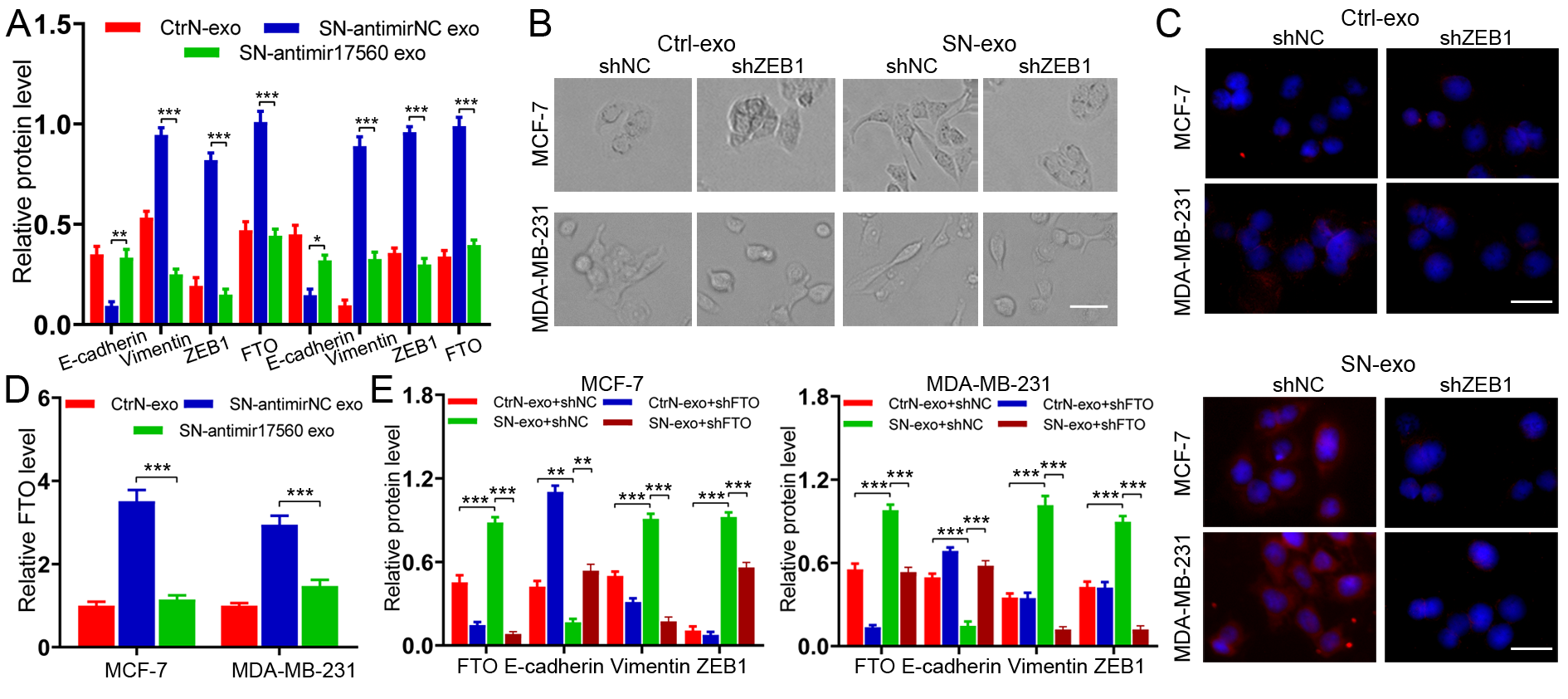

Supplement: Supplementary file 5 — Supplementary Figure 4 [file 41419_2022_5317_MOESM5_ESM.tif]

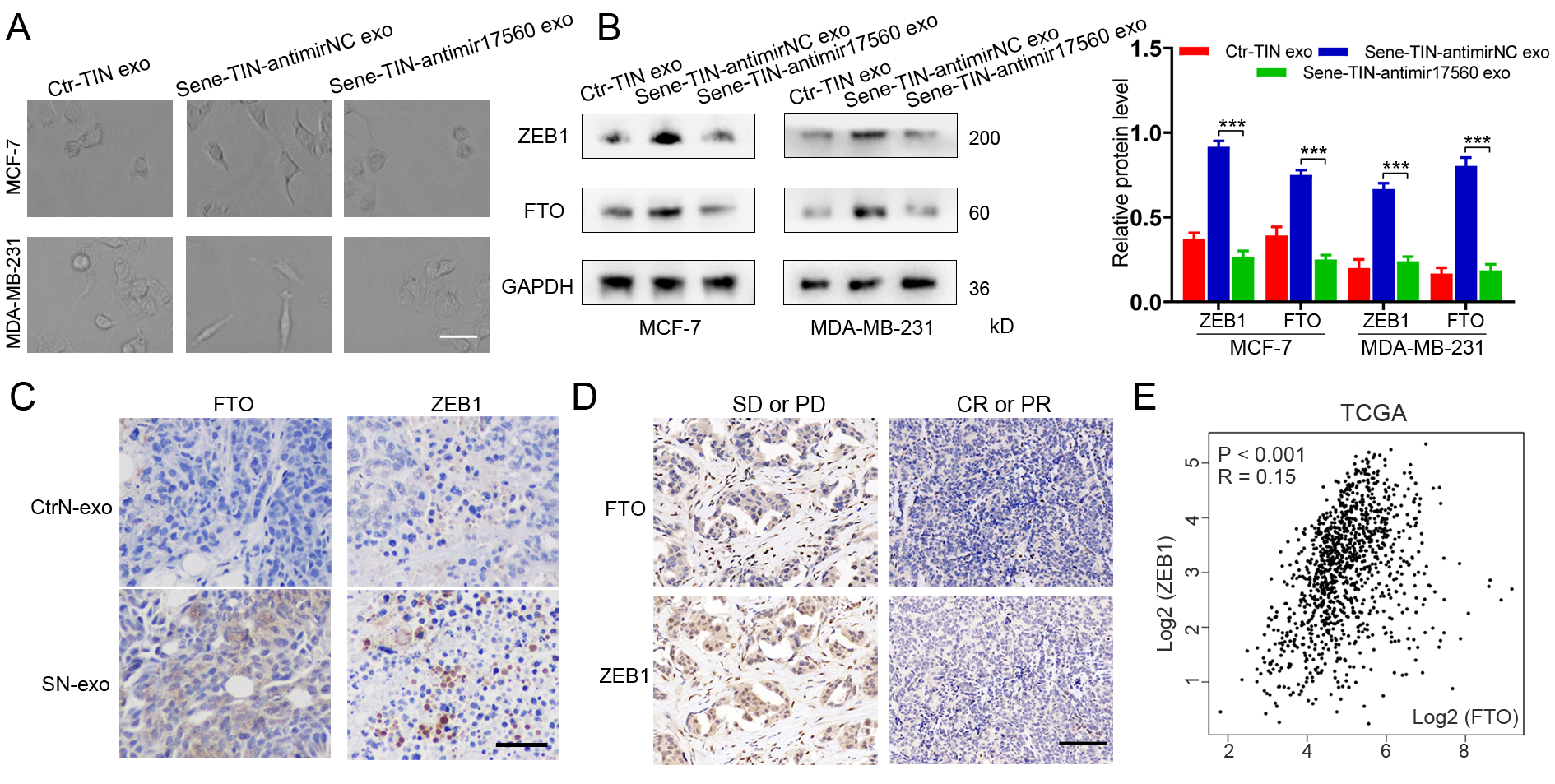

Supplement: Supplementary file 6 — Supplementary Figure 5 [file 41419_2022_5317_MOESM6_ESM.tif]

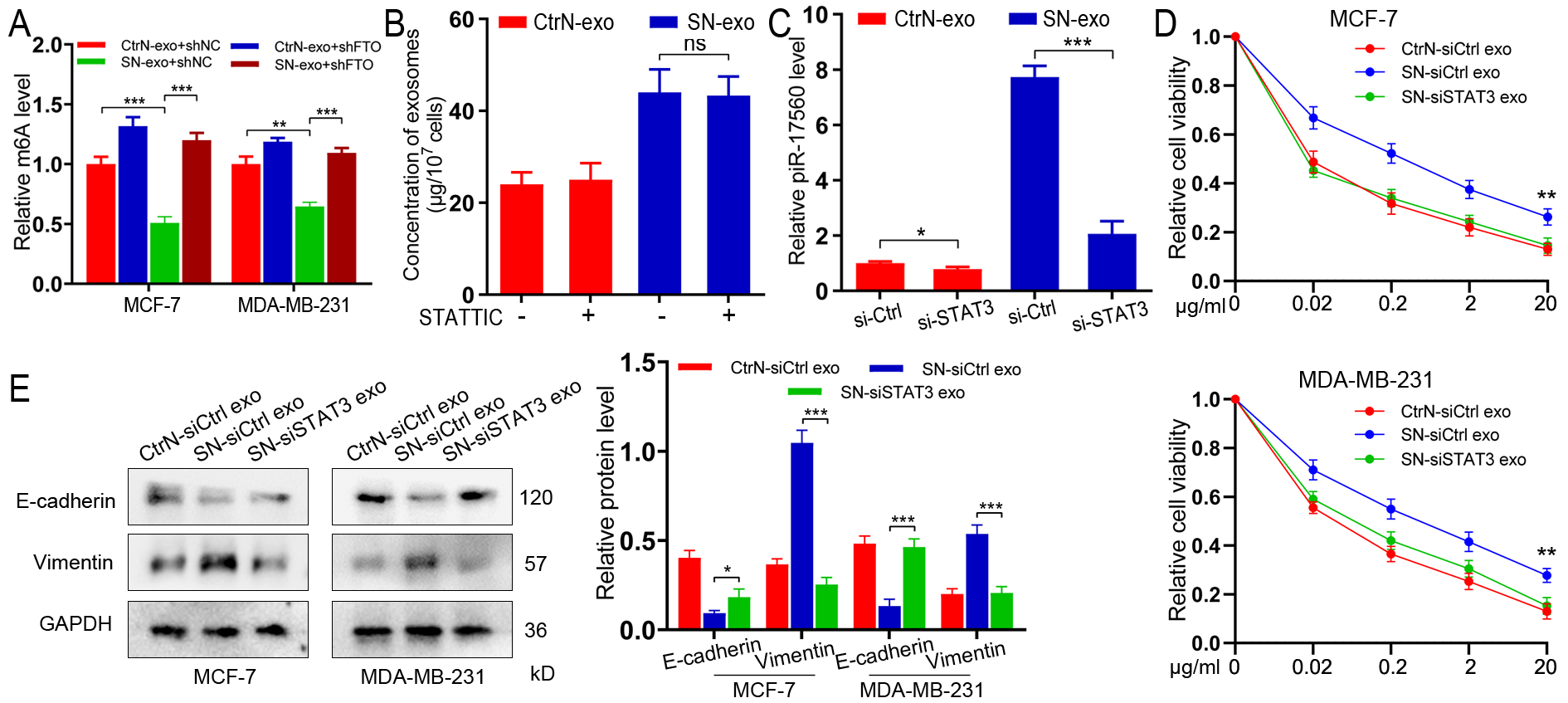

Supplement: Supplementary file 7 — Supplementary Figure 6 [file 41419_2022_5317_MOESM7_ESM.tif]

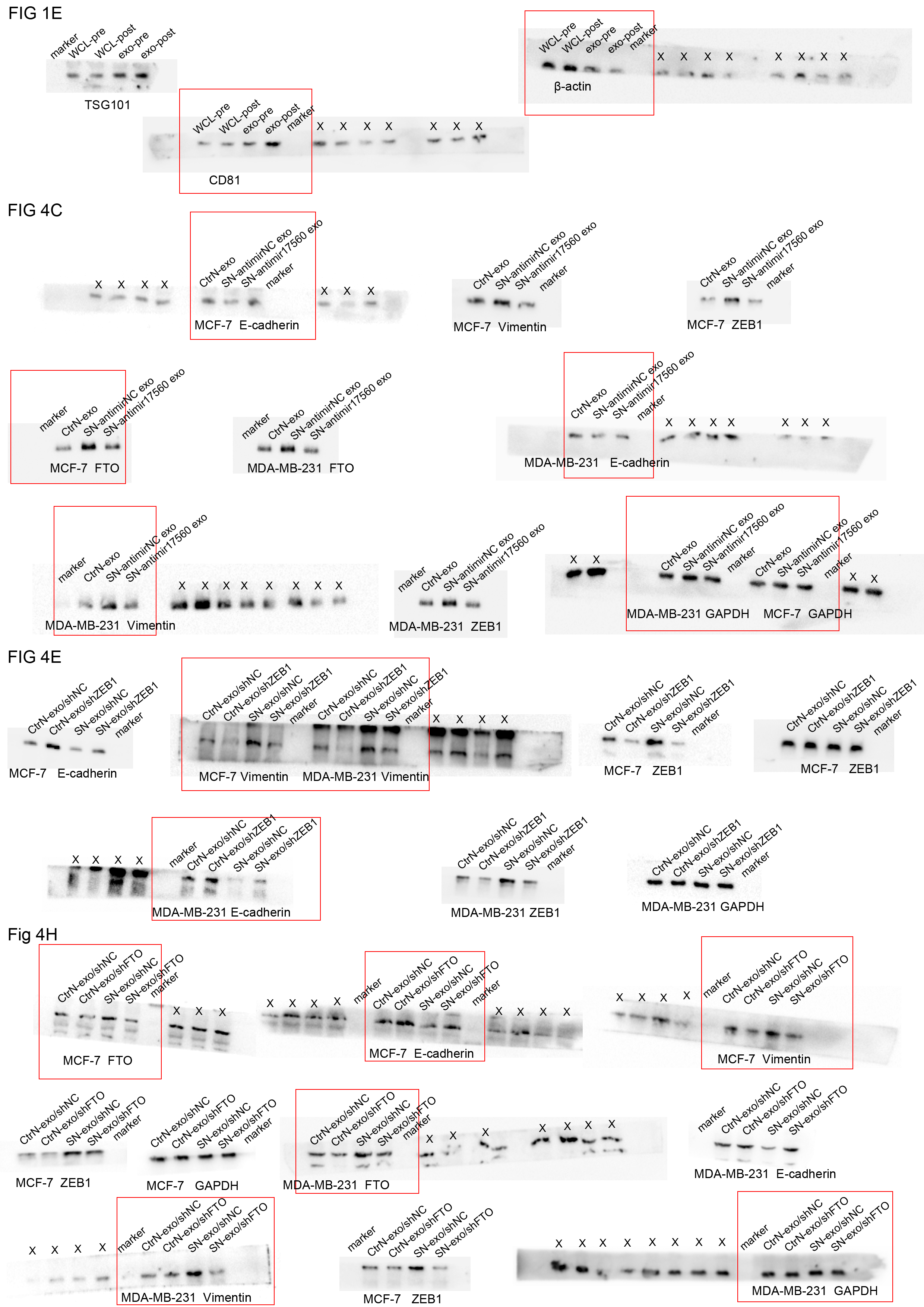

Supplement: Supplementary file 9 — Original Data File [file 41419_2022_5317_MOESM9_ESM.tif]

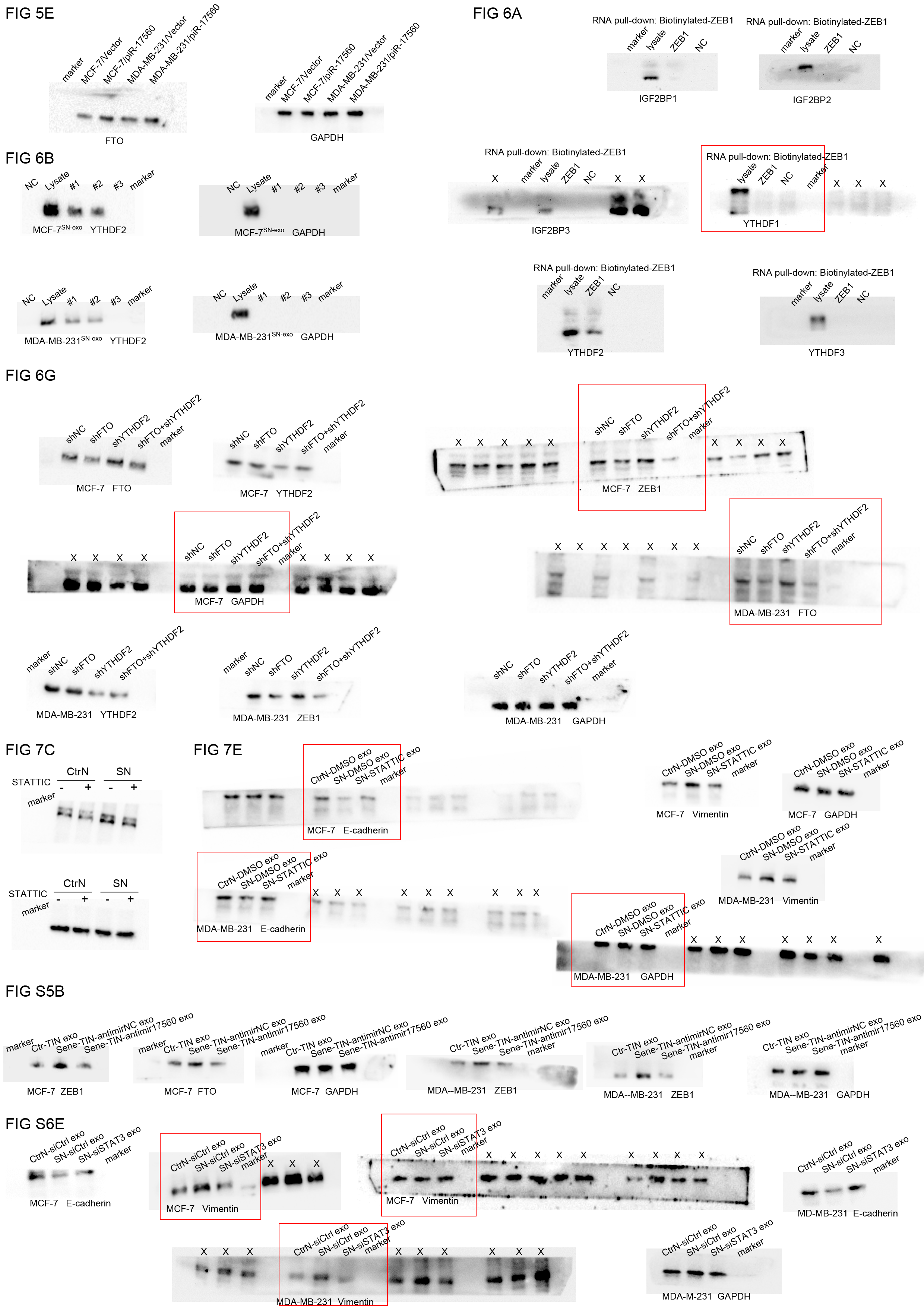

Supplement: Supplementary file 10 — Original Data File [file 41419_2022_5317_MOESM10_ESM.tif]
