# Supplementary material for: Senescent neutrophils-derived exosomal piRNA-17560 promotes chemoresistance and EMT of breast cancer via FTO-mediated m6A demethylation
Source: Cell Death Dis. 2022 Oct 27;13(10):905. doi: 10.1038/s41419-022-05317-3 (PMC9613690; doi:10.1038/s41419-022-05317-3)
Supplement: Supplementary file 8 — Supplementary Table [file 41419_2022_5317_MOESM8_ESM.docx]

**Table S1.** The differentially expressed genes in MCF-7 cells treated with senescent neutrophils-derived exosomes.

| Gene.symbol | P-value | logFC | Gene.symbol | P-value | logFC |
| --- | --- | --- | --- | --- | --- |
| PRKAR2B | 0.000483 | 3.31 | NPEPL1 | 0.004899 | -1.05 |
| ZEB1 | 0.004369 | 3.03 | MAVS | 0.006624 | -1.05 |
| NT5E | 0.000483 | 2.66 | ERAP1 | 0.01293 | -1.05 |
| RAB3B | 0.000527 | 2.48 | RNASE4 | 0.021607 | -1.05 |
| VIM | 0.000527 | 2.41 | ELMOD2 | 0.003381 | -1.06 |
| HRASLS | 0.001066 | 2.29 | CERS6 | 0.003634 | -1.06 |
| IL32 | 0.000859 | 2.24 | HSPB8 | 0.007218 | -1.06 |
| FHL1 | 0.000862 | 2.18 | JMJD1C | 0.003135 | -1.07 |
| RIN2 | 0.000723 | 2.08 | GGACT | 0.003446 | -1.07 |
| RBP1 | 0.001317 | 2 | LIPG | 0.003857 | -1.07 |
| TMSB15B | 0.002748 | 1.99 | CMTM4 | 0.005135 | -1.07 |
| TUBGCP3 | 0.000862 | 1.94 | MYSM1 | 0.005336 | -1.07 |
| TSPAN7 | 0.001406 | 1.93 | DOCK9 | 0.009474 | -1.07 |
| CD24 | 0.000954 | 1.89 | CRIPAK | 0.003518 | -1.08 |
| TMEM158 | 0.000812 | 1.87 | DZIP3 | 0.005574 | -1.08 |
| PALM2 | 0.001158 | 1.83 | CLCN5 | 0.00903 | -1.08 |
| LOC101929340 | 0.001155 | 1.8 | ANKRA2 | 0.003622 | -1.09 |
| KDM1B | 0.001829 | 1.78 | IER2 | 0.004498 | -1.09 |
| PPP1R2 | 0.003207 | 1.77 | RRP7A | 0.00734 | -1.09 |
| BIRC3 | 0.001567 | 1.73 | ERVMER34-1 | 0.003136 | -1.1 |
| BEX5 | 0.001353 | 1.72 | PHLDA1 | 0.003634 | -1.1 |
| RPS24 | 0.001039 | 1.68 | PHF14 | 0.004138 | -1.1 |
| LOC101927934 | 0.004518 | 1.67 | NUCKS1 | 0.005127 | -1.1 |
| FAM122C | 0.001446 | 1.63 | SYS1 | 0.022259 | -1.1 |
| HS3ST3B1 | 0.001669 | 1.61 | FNBP1L | 0.003504 | -1.11 |
| ESAM | 0.001438 | 1.59 | SLFN5 | 0.009211 | -1.11 |
| TLCD1 | 0.001353 | 1.56 | CXXC5 | 0.003105 | -1.12 |
| FTO | 0.001603 | 1.55 | TMCO3 | 0.003254 | -1.12 |
| CALM1 | 0.001208 | 1.52 | TFDP2 | 0.003673 | -1.12 |
| SERINC2 | 0.002101 | 1.51 | CIRBP | 0.004762 | -1.12 |
| GPRC5A | 0.001446 | 1.5 | INPP4B | 0.014381 | -1.12 |
| AGFG2 | 0.00903 | 1.48 | SRSF8 | 0.003041 | -1.13 |
| DNMBP | 0.001488 | 1.46 | SLC35B3 | 0.005209 | -1.13 |
| ARNTL2-AS1 | 0.001633 | 1.45 | ADAMTS17 | 0.013056 | -1.13 |
| COL6A1 | 0.00922 | 1.44 | PAN3 | 0.002573 | -1.14 |
| TM4SF5 | 0.002173 | 1.43 | RABGGTB | 0.002925 | -1.14 |
| ECM1 | 0.002364 | 1.42 | TRIB2 | 0.003983 | -1.14 |
| SCLY | 0.001627 | 1.41 | RAB40C | 0.01451 | -1.14 |
| LENG8 | 0.00344 | 1.4 | ATF3 | 0.002662 | -1.15 |
| RBM8A | 0.001781 | 1.39 | SNORA50C | 0.003384 | -1.15 |
| CTSS | 0.006411 | 1.39 | TNRC6B | 0.004277 | -1.15 |
| BMP2 | 0.016104 | 1.38 | VDR | 0.010749 | -1.15 |
| LINC00992 | 0.00285 | 1.37 | TRIM2 | 0.003518 | -1.16 |
| LPCAT1 | 0.0046 | 1.36 | SLC25A12 | 0.009135 | -1.16 |
| ITPKA | 0.00995 | 1.35 | SLC12A2 | 0.003645 | -1.17 |
| DNMBP | 0.002762 | 1.34 | C11orf1 | 0.004899 | -1.17 |
| RABL6 | 0.001575 | 1.33 | USH1C | 0.002662 | -1.18 |
| PLAUR | 0.003673 | 1.33 | NAV1 | 0.004138 | -1.18 |
| CTHRC1 | 0.002387 | 1.31 | NMNAT2 | 0.005577 | -1.18 |
| UPP1 | 0.001669 | 1.3 | ANK2 | 0.002412 | -1.19 |
| HLA-DRB1 | 0.001771 | 1.29 | CDK8 | 0.004131 | -1.19 |
| RELB | 0.007145 | 1.29 | ZRANB1 | 0.002268 | -1.2 |
| CYP24A1 | 0.005068 | 1.28 | PP7080 | 0.003526 | -1.2 |
| PAPSS2 | 0.009594 | 1.27 | XPNPEP3 | 0.004277 | -1.2 |
| PEX6 | 0.002499 | 1.26 | CDK8 | 0.002112 | -1.21 |
| GTPBP10 | 0.011718 | 1.26 | CIRBP | 0.003364 | -1.21 |
| SLC40A1 | 0.007498 | 1.25 | PPM1L | 0.019761 | -1.21 |
| SOX7 | 0.002503 | 1.23 | RIMKLB | 0.002548 | -1.22 |
| TTYH2 | 0.003504 | 1.23 | RASSF8-AS1 | 0.004345 | -1.22 |
| ACSL5 | 0.00538 | 1.22 | DNM3 | 0.002171 | -1.23 |
| HSP90B1 | 0.002345 | 1.21 | KLF11 | 0.006698 | -1.23 |
| BRD3 | 0.004074 | 1.21 | DUSP13 | 0.002631 | -1.24 |
| CDC42EP1 | 0.002257 | 1.2 | SLC16A10 | 0.002036 | -1.25 |
| SRM | 0.003956 | 1.2 | CCNB1IP1 | 0.002315 | -1.25 |
| PPP1R9B | 0.002335 | 1.19 | IGF1R | 0.005135 | -1.25 |
| AKAP12 | 0.003543 | 1.19 | ARRB1 | 0.002098 | -1.26 |
| VIMENTIN | 0.003785 | 1.19 | ALS2 | 0.002604 | -1.26 |
| CD55 | 0.005211 | 1.19 | SH3RF3 | 0.004205 | -1.26 |
| HIP1 | 0.020332 | 1.19 | SLC39A8 | 0.006486 | -1.26 |
| CHGB | 0.002912 | 1.18 | SMYD3 | 0.003504 | -1.27 |
| TPP1 | 0.005162 | 1.18 | PPFIBP1 | 0.002036 | -1.28 |
| BTBD19 | 0.016956 | 1.18 | H3F3A | 0.00435 | -1.28 |
| RBM24 | 0.004845 | 1.17 | PDP1 | 0.002567 | -1.29 |
| PTCD3 | 0.002413 | 1.16 | MBNL1 | 0.00176 | -1.3 |
| SAC3D1 | 0.003673 | 1.16 | CYP4V2 | 0.002674 | -1.3 |
| AXL | 0.003825 | 1.15 | ITGA2 | 0.034324 | -1.3 |
| EREG | 0.006058 | 1.15 | KDM7A | 0.005999 | -1.31 |
| KANSL1 | 0.015805 | 1.15 | SAMD4A | 0.002257 | -1.32 |
| ATP2A2 | 0.003634 | 1.14 | S100A10 | 0.023075 | -1.32 |
| SNAI1 | 0.008506 | 1.14 | ARRB1 | 0.002345 | -1.33 |
| DCLK1 | 0.003105 | 1.13 | ACSL6 | 0.003601 | -1.33 |
| TM2D1 | 0.004243 | 1.13 | CCDC3 | 0.001661 | -1.34 |
| RPS6KA2 | 0.005574 | 1.13 | FLRT3 | 0.006124 | -1.34 |
| SLC44A2 | 0.011865 | 1.13 | USF3 | 0.001893 | -1.35 |
| TSPAN5 | 0.002912 | 1.12 | LAMP3 | 0.009487 | -1.35 |
| CDH2 | 0.004182 | 1.12 | KBTBD7 | 0.003033 | -1.36 |
| CBX5 | 0.0053 | 1.12 | HMGB1 | 0.002144 | -1.37 |
| UGCG | 0.011887 | 1.12 | GSTM4 | 0.001544 | -1.38 |
| ADAMTSL2 | 0.003545 | 1.11 | VANGL1 | 0.003601 | -1.38 |
| ASB2 | 0.007194 | 1.11 | CLCN4 | 0.002771 | -1.39 |
| VGF | 0.012396 | 1.11 | GYG2 | 0.004452 | -1.4 |
| RTN1 | 0.002893 | 1.1 | FAM117B | 0.001606 | -1.41 |
| DCBLD2 | 0.003785 | 1.1 | ARRB1 | 0.001378 | -1.42 |
| TWIST1 | 0.006121 | 1.1 | KLF12 | 0.003169 | -1.42 |
| CNPY3 | 0.002886 | 1.09 | KIAA1211 | 0.004898 | -1.43 |
| NSUN4 | 0.003384 | 1.09 | ATP2C1 | 0.003536 | -1.44 |
| AAED1 | 0.004152 | 1.09 | RHOQ | 0.0014 | -1.45 |
| WSCD1 | 0.005253 | 1.09 | SNTB1 | 0.001221 | -1.46 |
| TRIM6 | 0.021043 | 1.09 | INSIG1 | 0.001704 | -1.47 |
| SNAI2 | 0.00373 | 1.08 | STRA6 | 0.007373 | -1.48 |
| RC3H2 | 0.006086 | 1.08 | TNFRSF10D | 0.001361 | -1.49 |
| GRIN2D | 0.014002 | 1.08 | COX3 | 0.025015 | -1.49 |
| MINK1 | 0.003384 | 1.07 | VCPIP1 | 0.001544 | -1.5 |
| ACTA2 | 0.003906 | 1.07 | SUN1 | 0.001268 | -1.51 |
| TPSAB1 | 0.004767 | 1.07 | PSG1 | 0.001268 | -1.52 |
| ETV1 | 0.053678 | 1.07 | DLEU2 | 0.001288 | -1.53 |
| PLOD1 | 0.003601 | 1.06 | LINC01003 | 0.001145 | -1.54 |
| TSHZ3 | 0.003854 | 1.06 | FAM117B | 0.00116 | -1.55 |
| RAB27A | 0.005386 | 1.06 | TNFSF10 | 0.002216 | -1.55 |
| TPM1 | 0.009264 | 1.06 | ZFAS1 | 0.001075 | -1.57 |
| TWIST2 | 0.032997 | 1.06 | PHF14 | 0.001218 | -1.57 |
| HNRNPA3 | 0.003904 | 1.05 | CDH11 | 0.001553 | -1.58 |
| ASMTL | 0.005435 | 1.05 | SYK | 0.001734 | -1.61 |
| PEAR1 | 0.012244 | 1.05 | MUC16 | 0.007961 | -1.61 |
| YWHAE | 0.003808 | 1.04 | NEDD9 | 0.006573 | -1.62 |
| EIF3B | 0.005308 | 1.04 | ARHGEF9 | 0.001344 | -1.64 |
| PILRB | 0.011609 | 1.04 | KCNJ5 | 0.003389 | -1.66 |
| PRPF4 | 0.003656 | 1.03 | HIST1H2BG | 0.000988 | -1.68 |
| FSCN1 | 0.004226 | 1.03 | TACSTD2 | 0.001339 | -1.69 |
| GALT | 0.006906 | 1.03 | RNF217 | 0.00135 | -1.7 |
| TMEM141 | 0.003641 | 1.02 | TRIM73 | 0.001155 | -1.72 |
| COL13A1 | 0.00528 | 1.02 | ESRP1 | 0.000954 | -1.73 |
| TPM2 | 0.006991 | 1.02 | PDE6D | 0.000862 | -1.74 |
| AHNAK | 0.012681 | 1.02 | LRP8 | 0.001172 | -1.75 |
| TRIM14 | 0.016236 | 1.02 | OTUD6B-AS1 | 0.000859 | -1.77 |
| SIK3 | 0.004226 | 1.01 | MDM2 | 0.001264 | -1.78 |
| CLN5 | 0.006573 | 1.01 | CLU | 0.001149 | -1.82 |
| FAAP20 | 0.012504 | 1.01 | JUN | 0.001208 | -1.84 |
| PTPN13 | 0.036167 | 1.01 | ADAMTS17 | 0.003268 | -1.85 |
| RHOQ | 0.003785 | -1.01 | PCDH9 | 0.003188 | -1.87 |
| LOC399900 | 0.004206 | -1.01 | BHLHE41 | 0.000862 | -1.91 |
| SERPINB9 | 0.005274 | -1.01 | KLHL24 | 0.001313 | -1.93 |
| PTPRE | 0.006976 | -1.01 | NEAT1 | 0.071423 | -1.94 |
| ZNF292 | 0.024702 | -1.01 | STRA6 | 0.001107 | -1.95 |
| NCOA2 | 0.004206 | -1.02 | PSMB4 | 0.000862 | -1.98 |
| ECHDC1 | 0.004849 | -1.02 | CDH1 | 0.002503 | -1.99 |
| FAM120AOS | 0.005812 | -1.02 | MCTP1 | 0.002358 | -2.06 |
| MTX3 | 0.008475 | -1.02 | CTNNA1 | 0.00069 | -2.09 |
| EEF1D | 0.027031 | -1.02 | LOC100506922 | 0.000674 | -2.12 |
| SLC36A4 | 0.003869 | -1.03 | METTL9 | 0.004822 | -2.15 |
| CENPL | 0.004577 | -1.03 | EDN3 | 0.001544 | -2.2 |
| FAM171B | 0.005168 | -1.03 | TNFSF10 | 0.000798 | -2.24 |
| LOC103344931 | 0.006906 | -1.03 | CXCL14 | 0.000862 | -2.32 |
| DLEU2 | 0.018837 | -1.03 | INSIG1 | 0.000687 | -2.4 |
| RBM15 | 0.004138 | -1.04 | C6orf62 | 0.000954 | -2.52 |
| FAM214A | 0.005162 | -1.04 | RBM3 | 0.000527 | -2.56 |
| ZCCHC14 | 0.007397 | -1.04 | ETNK1 | 0.000859 | -2.68 |
| RPL18A | 0.101683 | -1.04 | ADGRG2 | 0.000507 | -3.1 |
| CDC42EP3 | 0.003373 | -1.05 | KCNJ5 | 0.000321 | -3.86 |
| CRIPT | 0.004182 | -1.05 |  |  |  |

**Table S2**. The specific sequences of wild-type or m6A motif mutant ZEB1 3’UTR.

| Vectors | Sequence* (5’→3’) |
| --- | --- |
| ZEB1 3’UTR  wild-type | ACTGAAACACTG**GGACA**TTTCATCCTTC……ATTTCAGACAT**GGACA**TGCTATTGTT |
| ZEB1 3’UTR  mutation | ACTGAAACACTG**GGTCA**TTTCATCCTTC……ATTTCAGACAT**GGTCA**TGCTATTGTT |

*The sequence was inserted to the vectors, and the m6A motif sites were bolded and underline.

**Table S3.** Primers used in this study.

| Gene symbol | Sequences (5’→3’) | |
| --- | --- | --- |
| ZEB1 | sense | TTACACCTTTGCATACAGAACCC |
|  | antisense | TTTACGATTACACCCAGACTGC |
| FTO | sense | ACTTGGCTCCCTTATCTGACC |
|  | antisense | TGTGCAGTGTGAGAAAGGCTT |
| GAPDH | sense | CTGGGCTACACTGAGCACC |
|  | antisense | AAGTGGTCGTTGAGGGCAATG |
| CISH | sense | GAACTGCCCAAGCCAGTCAT |
|  | antisense | GCTATGCACAGCAGATCCTCC |
| IL6 | sense | ACTCACCTCTTCAGAACGAATTG |
|  | antisense | CCATCTTTGGAAGGTTCAGGTTG |
| IL6R | sense | CCCCTCAGCAATGTTGTTTGT |
|  | antisense | CTCCGGGACTGCTAACTGG |
| JAK1 | sense | CTTTGCCCTGTATGACGAGAAC |
|  | antisense | ACCTCATCCGGTAGTGGAGC |
| JAK2 | sense | ATCCACCCAACCATGTCTTCC |
|  | antisense | ATTCCATGCCGATAGGCTCTG |
| JAK3 | sense | CCTGATCGTGGTCCAGAGAG |
|  | antisense | GCAGGGATCTTGTGAAATGTCAT |
| PIAS3 | sense | GGTTTGAGGAAGCGCACTTTA |
|  | antisense | CTCCTGGCAGAACCTCTCT |
| PTPRU | sense | CGGGCGTCTCTAACTTCGC |
|  | antisense | GGAGTTGGTGTTGAGCTGGA |
| REG1A | sense | ACCAGCTCATACTTCATGCTGA |
|  | antisense | CCAGGTCTCACGGTCTTCAT |
| SRC | sense | GACAGGCTACATCCCCAGC |
|  | antisense | CGTCTGGTGATCTTGCCAAAA |
| STAT3 | sense | ACCAGCAGTATAGCCGCTTC |
|  | antisense | GCCACAATCCGGGCAATCT |

**Table S4.** Primers used for stem-loop RT-PCR of piRNAs.

| Gene symbol | Stem-loop Reverse transcription primers (5’→3’) | Sense primer (5’→3’) | Antisense primer (5’→3’) |
| --- | --- | --- | --- |
| piR-17560 | GTCGTATCCAGTGCGTGTCGTGGAGTCGGCAATTGCACTGGATACGATCTCAGGTA | GCGATGGATATGATGACTGATT | CAGTGCGTGTCGTGGAGT |
| piR-805 | GTCGTATCCAGTGCGTGTCGTGGAGTCGGCAATTGCACTGGATACGAACCTGGCTG | GCGAACCTAGGACTTGACCAAG | CAGTGCGTGTCGTGGAGT |
| piR-17033 | GTCGTATCCAGTGCGTGTCGTGGAGTCGGCAATTGCACTGGATACGAGTACACGCA | GCGATGGAATGGAAACATTTAT | CAGTGCGTGTCGTGGAGT |
| U6 | AACGCTTCACGAATTTGCGT | CTCGCTTCGGCAGCACA | AACGCTTCACGAATTTGCGT |

**Table S5.** Sequences of shRNA and siRNA against specific target in this study.

| Symbol | Sequences (5’→3’) | |
| --- | --- | --- |
| RAB27A siRNA | sense | GCUUAACGACAGCGUUCUUTT |
|  | antisense | AAGAACGCUGUCGUUAAGCTT |
| RAB27B siRNA | sense | GCCAUGGGCUUCUUAUUAATT |
|  | antisense | UUAAUAAGAAGCCCAUGGCTT |
| STAT3 siRNA | sense | UUUUGAGUGGGUAUCAACCAG |
|  | antisense | GGUUGAUACCCACUCAAAAAG |
| Ctrl siRNA | sense | UUCUCCGAACGUGUCACGUTT |
|  | antisense | ACGUGACACGUUCGGAGAATT |
| Symbol | Targeting sequences (5’---3’) | |
| shFTO | TCACGAATTGCCCGAACATTA | |
| ShZEB1 | CCTCTCTGAAAGAACACATTA | |

**Table S6.** Primers for construction of luciferase reporter plasmids.

| Primers for FTO 3’UTR construct | Primer sequences (5’→3’) | Enzyme |
| --- | --- | --- |
| sense: | TATAGCTCAGCACACGGGAAGGAGAT | Blpl |
| antisense: | GGCTCGAGTCTCAACACGATGAGAC | Blul |
| Primers for ZEB1 3’UTR construct |  |  |
| sense: | CGCTCTAGAAGGTAGTAATTTCTAA | Xbal |
| antisense: | TATACCCGGGATAACACTGTTAAATCA | Hpal |
