# Supplementary material for: Senescent neutrophils-derived exosomal piRNA-17560 promotes chemoresistance and EMT of breast cancer via FTO-mediated m6A demethylation
Source: Cell Death Dis. 2022 Oct 27;13(10):905. doi: 10.1038/s41419-022-05317-3 (PMC9613690; doi:10.1038/s41419-022-05317-3)
Supplement: Supplementary file 12 — Author Contribution Form [file 41419_2022_5317_MOESM12_ESM.pdf]

DECLARATION OF CONTRIBUTIONS TO ARTICLE

ADMC

Manuscript Number:	Journal Name:	
CDDIS-22-1840	*Cell Death & Disease*	(the 'Journal')

Proposed Title of the Contribution:

Senescent neutrophils-derived exosomal piRNA-17560 promotes chemoresistance and EMT of breast cancer via FTO-mediated m6A demethylation (the 'Contribution)

Author(s):

Baochi Ou, Yuan Liu, Zongxuan Gao, Jun Xu, Yunwen Yan, Yongxiang Li, Jingjie Zhang (the 'Authors')

For all *CDDis* articles, each person named as an author in the published version must be able to show he or she has contributed substantially to the article.

Authorship credit should be based on 1) substantial contributions to conception and design, acquisition of data, or analysis and interpretation of data; 2) drafting the article or revising it critically for important intellectual content; and 3) final approval of the version to be published. Authors should meet conditions 1, 2 and 3.

Any person who cannot be shown to have made a substantial contribution to the article cannot be listed as an author in the final version. The name of any person who is deemed to have made a minor contribution can, however, appear in the Acknowledgments section of the article.

Please complete the table below to indicate the contributions of all named authors to the manuscript.

Author Full Name:	Specification of Contribution to the Manuscript:
Baochi Ou	conception and design, acquisition of data, or analysis and interpretation of data, final approval of the version to be published
Yuan Liu	acquisition of data, drafting the article, final approval of the version to be published
Zongxuan Gao	acquisition of data, final approval of the version to be published
Jun Xu	analysis and interpretation of data, final approval of the version to be published
Yunwen Yan	acquisition of data, final approval of the version to be published
Yongxiang Li	conception and design, final approval of the version to be published
Jingjie Zhang	conception and design, final approval of the version to be published

Please complete the table below to indicate the contributions of all named authors to the figures.

Figure 1:

Baochi Ou, Yuan Liu, Zongxuan Gao and Jingjie Zhang generated the data, Baochi Ou assembled the figure

Figure 2:

Yuan Liu, Zongxuan Gao, Jun Xu and Yunwen Yan generated the data, Baochi Ou assembled the figure

Figure 3:

Yuan Liu, Yongxiang Li and Jingjie Zhang generated and analyzed the data, Baochi Ou assembled the figure

Figure 4:

Baochi Ou, Yuan Liu, Zongxuan Gao and Yongxiang Li generated the data, Baochi Ou assembled the figure

Figure 5:

Yuan Liu, Jun Xu and Yunwen Yan generated the data, Baochi Ou assembled the figure

Figure 6:

Baochi Ou, Jingjie Zhang and Yongxiang Li generated the data, Baochi Ou assembled the figure

Signed for and on behalf of the Author(s):	Print Name:	Date:
Baochi Ou.	Baochi Ou	2022-5-21
